# Digital adaptation of the clinically effective REACH-HF home-based cardiac rehabilitation programme for people living with heart failure (D:REACH-HF)

**DOI:** 10.1177/20552076251406545

**Published:** 2025-12-18

**Authors:** Samantha B van Beurden, Rosina Cross, Sinéad T J McDonagh, Liz Clark, Chloe Thomas, Colin J Greaves, Patrick Doherty, Rod S Taylor, Hasnain M Dalal

**Affiliations:** 1 Department of Health and Community Sciences, 171002 University of Exeter Medical School, Faculty of Health and Life Sciences, 3286 University of Exeter, Exeter, UK; 2 3286 Public Contributor (PPI), University of Exeter, Exeter, UK; 3 Psychology, 3286 University of Exeter, Exeter, UK; 4 School of Sport, Exercise & Rehabilitation Sciences, 1724 University of Birmingham, Birmingham, UK; 5 Health Sciences, 8748 University of York, York, UK; 6 School of Health and Wellbeing, 3526 University of Glasgow, Glasgow, UK; 7 8028 Royal Cornwall Hospitals Trust, Truro, UK

**Keywords:** Cardiac rehabilitation, heart failure, patient engagement, Person-Based Approach, asynchronous rehabilitation, digital intervention, qualitative research, digital health, caregivers, telehealth, self-management, human support

## Abstract

**Background:**

Despite strong recommendations for heart failure (HF) rehabilitation, participation (uptake and sustained engagement) remains low. Digital interventions may enhance participation and scalability, yet evidence for feasibility in real-world National Health Service settings remains limited. Rehabilitation Enablement in Chronic Heart Failure (REACH-HF), an effective home-based HF cardiac rehabilitation programme, is currently delivered using paper-based manuals with facilitator support.

**Objectives:**

To co-develop and assess the feasibility of a digitally adapted and enhanced version of REACH-HF (D:REACH-HF) with patients, caregivers, and healthcare professionals.

**Methods:**

Following the Person-Based Approach, D:REACH-HF was iteratively co-developed with patients and caregivers in a public involvement group (stage 1) and through qualitative research (stages 2A and 2B). Usability and acceptability of content and design iterations were evaluated with patients, caregivers, and healthcare professionals through think-aloud interviews (*n* = 20 participants) and feasibility of the fully-functional intervention with facilitation, was assessed with semi-structured interviews at 2–4 and 10–12 weeks (*n* = 10).

**Results:**

Participants rated D:REACH-HF highly (mean app quality score: 4.02/5), particularly for information credibility and functionality. Key benefits included flexibility, structured self-monitoring, and healthcare professional support. Remote access to patient data enabled more efficient consultations, allowing healthcare professionals to focus on tailoring their support to patient needs. Challenges included technical issues, digital literacy, and engagement variability. All patients requested continued access to the platform, highlighting perceived long-term value.

**Conclusion:**

The D:REACH-HF programme is acceptable to patients and healthcare professionals. Moreover, as indicated by participant reports, it enables and achieves the same perceived benefits as the paper-based REACH-HF. However, evaluation of clinical and cost-effectiveness, implementation, and optimisation of D:REACH-HF for patients from under-researched and underserved communities is needed.

## Background

Currently, ∼64 million people live with heart failure (HF) globally and the prevalence is increasing.^1^ In England, the number of people with HF is projected to rise by 92%, to 2 million by 2040.^2^ Despite national and international guidance recommending cardiac rehabilitation (CR) for patients with HF,^3–5^ participation remains poor, with less than 20% uptake reported in the USA and Europe.^6,7^ This is even lower in the UK where only 13% of patients take up CR.^8^ Policy makers have set ambitious targets for increasing CR uptake among all eligible patients with cardiovascular disease to 70% in the USA^9^ and 85% (33% for HF) in the UK by 2028.^10^

Acceptability of group-based exercise and transport requirements for attending classes are key barriers to patient engagement^11,12^ contributing to a low uptake of traditional centre-based CR. To overcome such barriers, the National Institute for Health and Care Excellence (NICE) and the British Heart Foundation (BHF) are keen to promote ‘an expansion of new models of delivery including digitally-supported, home-based and more personalised “menu-based” approaches’,^13^^(p14)^ echoing patient voices.

Delivering CR digitally to improve access is gaining interest, with use of the internet in people aged over 65 years rising, web-based rehabilitation is being advocated as ‘one of the innovations required to future proof CR’.^14^^(p7)^ A 2018 systematic review concluded that tele-rehabilitation for cardiac patients is safe, feasible, and associated with high levels of adherence.^15^ Moreover, a recent Cochrane review showed that home-based CR (including digital/telehealth) and centre-based CR can have similar effects on clinical and health-related quality of life outcomes.^16^ Although digital platforms have previously been developed for managing HF,^17^ there are no robust peer-reviewed evaluations to date based in the UK.^16^

A possible solution to address this current unmet need is to offer digitally-adapted versions of existing evidence-based programmes. Rehabilitation Enablement in Chronic Heart Failure (REACH-HF) is a theory-based, healthcare professional facilitated, home-based CR programme using paper-based manuals, exercise videos, and relaxation audio, for people with HF and their caregivers. It was co-developed with patients, caregivers and CR healthcare professionals^12^ and has been shown to be effective and cost-effective in a multi-site randomised controlled trial.^18,19^ Learnings from this clinical trial, the caregiver assessments,^20^ mixed-methods process evaluation,^21^ and subsequent engagement with REACH-HF facilitators from the trial informed further refinements. REACH-HF has since been implemented widely in the National Health Service (NHS), with over 800 NHS staff trained to deliver the programme and continued research with initial adopter sites in the UK has provided insights to further improve implementation, delivery, and uptake.^22–24^

### Aims and objectives

The aim of this project was to co-develop an NHS-ready digitally-enhanced version of the clinically- and cost-effective REACH-HF programme^18,19^ (D:REACH-HF) with patients, caregivers, and healthcare professionals trained as REACH-HF facilitators.

Our objectives were to:

1. leverage the opportunities that digital technologies provide to:
  - ensure the accurate delivery of the existing programme, such that the content and core principles of REACH-HF remain the same, while adapting to a digital format (*adapt*), and
  - optimise the facilitation and caregiver engagement aspects of the REACH-HF programme (*enhance*).
2. Optimise for NHS-readiness by examining the feasibility of D:REACH-HF and identifying refinement targets to support integration into routine NHS services.

## Methods

### Design overview

**Figure 1.**
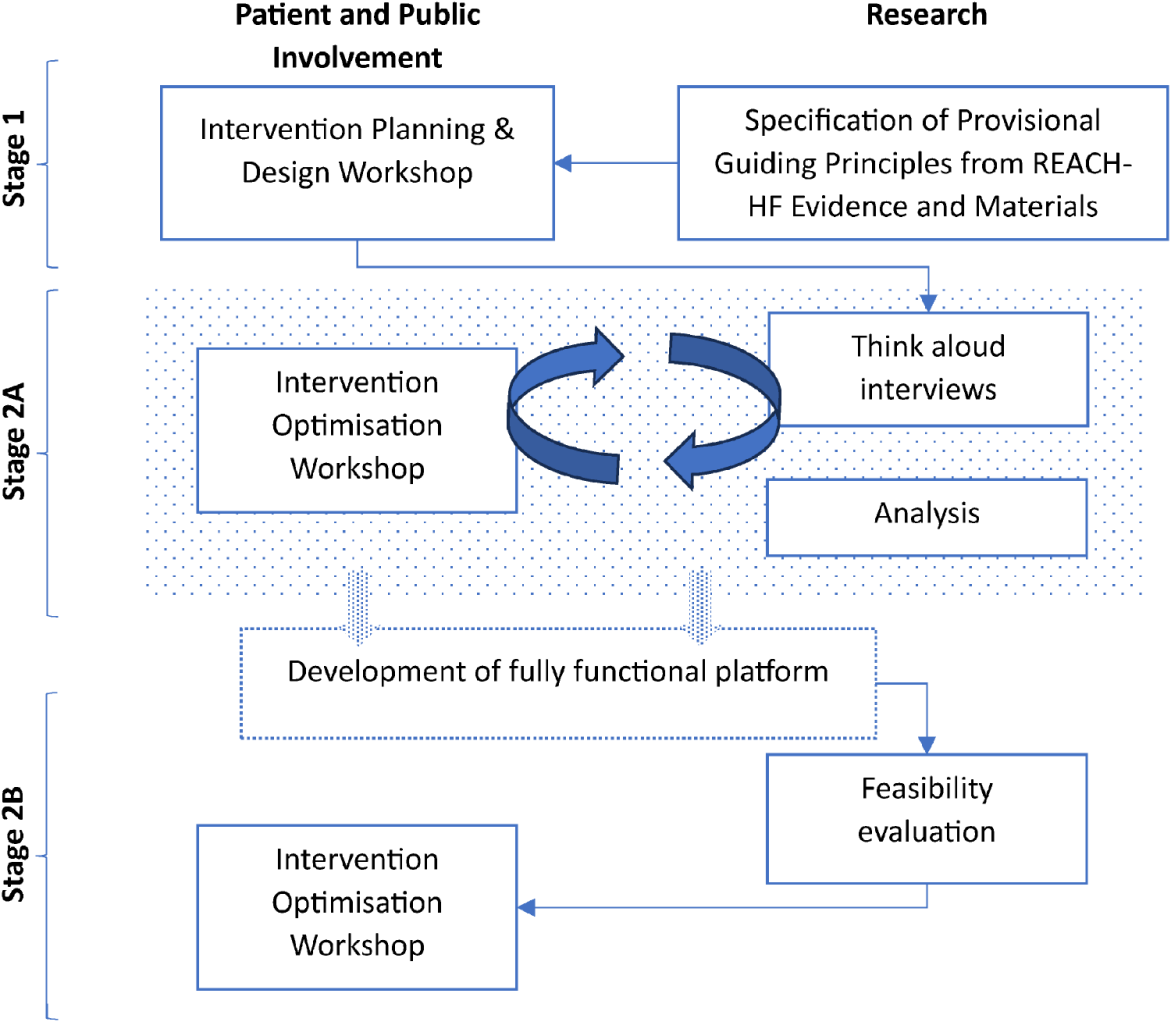
Study design overview.

This project was informed by the Medical Research Council's (MRC) Framework for complex interventions.^25^ We followed the Person-Based Approach,^26,27^ which has been shown to be a robust, systematic, and evidence-based approach^28–31^ to ensure that the development of the digitally-enhanced REACH-HF programme was thoroughly rooted in the context of living with HF, and as supporting and working with patients with HF. We conducted the first two stages of the Person-Based Approach (REF)^26,27^: (1) the intervention planning and design stage and (2) the optimisation stage. The latter was conducted over two consecutive phases using qualitative research, and involvement from a patient and public involvement (PPI) group throughout (see Figure 1).^32^

Our reporting follows guidance from three reporting guidelines: (1) guidance for the reporting of health intervention development studies (GUIDED),^33^ (2) a template for better reporting of intervention descriptions (TIDieR),^34^ and (3) standards for reporting of qualitative research studies (SRQR).^35^ All completed checklists are provided in the Supplemental file.

### PPI group

Involvement was fully integrated into this project as can be seen in Figure 1. Individuals were invited to join the PPI group via advertisements through the BHF, a patient-led HF charity (Pumping Marvellous) and X (formerly Twitter). A group of eight individuals (five living with HF, one caregiver, and two who had family members with HF; 50% female) from four regions of England met remotely via video-conferencing, five times, facilitated by SMcD and RC (November 2020 to January 2022) to: discuss and review the guiding principles, co-develop prototypes, discuss research findings, and, through problem-solving, generate data-driven refinements to the intervention.

### Stage 1: intervention planning and design

During the planning stage, the intervention development group (authors SBvB, RC, SMcD, CG, and two members from a healthcare software development company, Health and Care Innovation Ltd) used the findings from the REACH-HF process evaluation^21^ and REACH-HF intervention materials,^18^ to specify provisional guiding principles^26^ to complement the original intervention map and logic model, which links theory- and evidence- to desired behaviour changes, intervention components, and intended outcomes.^12^ Final guiding principles were used by the intervention developers to create mock-ups of D:REACH-HF using content from the paper-based REACH-HF manuals.

#### PPI activities

The provisional guiding principles were discussed, refined, and added to by the PPI group. The early-stage designs and sketches were discussed with the PPI group. Using PowerPoint and video-conferencing, the PPI group were able to actively input into the design by guiding the group facilitators to (in real time) change icons, move buttons, and alter layouts, thus co-developing the platform.

### Stage 2A: intervention optimisation

The first phase of stage 2 was conducted to optimise the draft intervention design and materials in relation to usability and anticipated ability to engage users and support behaviour change in preparation of the coding of the fully functional digital intervention. This stage consisted of cycles of data collection, analysis, and refinement (Figure 1). Think-aloud interviews (see Data and procedures) were conducted with research participants to test the usability and acceptability of the materials, data were analysed, and revised materials were tested in further think-aloud interviews. This stage received ethical approval from the North East – Newcastle & North Tyneside 1 Research Ethics Committee on 14/01/2021 (reference: 21/NE/0032).

#### Participants

Participant characteristics are described in Table 1. Twenty participants were recruited through NHS CR services, social media, and patient charity newsletters. Our sample consisted of 10 patients living with HF, one caregiver, and nine facilitators involved in the delivery of the (paper-based) REACH-HF intervention. All facilitators had experience in delivering centre- and home-based CR.

**Table 1.** Participant characteristics.

	Patients and caregivers (*n* = 9^a^)	REACH-HF facilitators (*n* = 6^a^)
Mean age in years (SD)	65.4 (7.6); range 50–74	50.1 (8.04); range 37–62
Female, *n* (%)	3 (33%)	6 (100%)
White ethnicity, *n* (%)	8 (89%)	6 (100%)
Highest education level		
A-level equivalent or higher, *n* (%)	9 (100%)	
Professional qualifications and higher, *n* (%)		6 (100%)

REACH-HF: Rehabilitation Enablement in Chronic Heart Failure.

a Only 15 questionnaires with demographic data were returned (one patient and their caregiver and three facilitators did not return questionnaires).

#### Data and procedures

Following written informed consent, each participant took part in up to two remote think-aloud interviews conducted via video-conferencing. Think-aloud interviews involved participants viewing and working through different iterations of the D:REACH-HF materials and being prompted to say out loud everything they thought about the materials and operation of the platform, encouraging them to ‘talk through’ and explain their responses and reactions. Interviews were audio-recorded and transcribed verbatim. Video-recordings of the interviews enabled clarification of exactly which screens, content, and functionality related to particular comments. The researcher took notes of any clear barriers to usability and engagement to enable rapid design changes in preparation for subsequent think-aloud interviews.

Think-aloud interviews and PPI group workshops continued until no new changes were suggested. The intervention development group then compiled the fully functional D:REACH-HF platform by adding and adapting all the content from the REACH-HF paper manuals, exercise videos, and relaxation audio.

#### Data analysis

The researcher worked through each transcript, line-by-line, coding any aspects of the data that pertained to (a) perceptions of the D:REACH-HF intervention or (b) perceived barriers to engagement with D:REACH-HF. Comments were added to a Table of Changes. We applied the Person-Based Approach's coding framework^36^ to the resulting Table of Changes. This framework includes codes such as REPeatedly mentioned (for anything that repeatedly came up in think-aloud interviews), EXPerience (for anything that was brought up by the PPI group), and IMPortant (for behaviour change). Using this framework enabled us to decide how important the proposed changes were, and why. The intervention development group then applied the ‘Must have, Should have, Could have, Would like to have’ categorisation process to determine which changes would be implemented, with decisions based on importance and feasibility, as well as available resources.

**Table 2.** Guiding principles for adaptation of REACH-HF to a digital format.

Design objective	Intervention features
Minimising cognitive load/overwhelm.	Clear and simple layout, language, and navigation procedures. Options to print or save key information or instructions wherever possible. Refer to existing non-digital sources of advice or support where appropriate, including the REACH-HF facilitator and peer or family support if possible.
Catering for heterogeneous population in terms of needs, preferences, and capabilities.	Tailoring of content to offer options for graded intensity of exercise and types of activities, with steer towards those most likely to be beneficial for user (based on initial consultation assessed need and capability, e.g. exercise capacity, misconceptions about HF, and anxiety and depression). Provision of carefully graded activities with very gradual increases from low activity baseline and addressing concerns and barriers for those lacking confidence or capability. Options of digital accessibility (high contrast, colour schemes, and font size).
Clear indication of when actions are required.	Text-based nudges towards help-seeking behaviour based on data-entered in the Progress Tracker regarding exercises, mood and well-being, and symptom following an algorithm implementing the key criteria in the paper-based Traffic Light Action Plan.
Minimising human error in data entry.	Review of data entry prior to saving submissions.
Enabling Family and Friends to support the patient with HF even when they are not co-habitants.	Family and Friends account created upon invite from the patient's account. Family and Friend (read-only) access to the HF manual, the family and friends resource, and the patient's Progress Tracker.
Enabling efficient personalised remote synchronous and asynchronous consultation following the REACH-HF ‘7-Steps of Facilitation’.	Healthcare professional access to the patient's progress Tracker data, summarising: Exercise goals and adherence Mood and well-being Medication adherence Symptom monitoring Healthcare professional access to notes and queries left by the patient. Options of viewing progress over time via either a quick glance or in-depth view of the elements above.
Working with CR service-based staff and patient management workflows.	Restricted permissions regarding creating, managing, and deleting patient and staff accounts. Options of different levels of user-accounts at the CR service: admin, facilitator, management, to enable different permissions/restrictions in functions and access.

REACH-HF: Rehabilitation Enablement in Chronic Heart Failure; HF: heart failure; CR: cardiac rehabilitation.

#### PPI activities

Items from the Table of Changes were presented to the PPI group over several workshops. The researchers (SBvB, RC, SMcD, CG) and the PPI group considered the barriers identified alongside any possible solutions, as to whether they were: (a) likely to impact behaviour change and (b) appropriate for the target population. These solutions were then added to the Table of Changes. In these workshops data-driven refinements were then developed which were tested in subsequent think-aloud interviews.

### Stage 2B: intervention optimisation

The second phase of stage 2 was conducted to assess feasibility of the full functional digital intervention and to inform further optimisation of engagement with the intervention, self-care behaviours, and potential self-reported outcomes. This stage received ethical approval from the East Midlands – Nottingham 2 Research Ethics Committee on 29/10/2021 (reference: 21/EM/0273).

#### Participant eligibility criteria

To get the perspectives from a range of different intended users, we invited patients, caregivers, and healthcare professionals to take part. Patients (and their caregivers) were eligible for D:REACH-HF if they had been referred to one of the four participating NHS CR services in the UK because they had HF, were eligible to receive local CR as assessed by their own clinical staff, were being offered REACH-HF in their service, and had access to an internet-connected digital device (e.g. laptop, tablet, or smartphone).

Healthcare professionals at the four NHS sites were eligible if they had completed the REACH-HF facilitator training from the Heart Manual Department at NHS Lothian and had been routinely delivering the programme in their service.

#### Data and procedures

Eleven REACH-HF-trained healthcare professionals from four NHS CR services in England (*n* = 3) and Scotland (*n* = 1) who were already routinely delivering REACH-HF, were trained to facilitate the use of D:REACH-HF. The training was delivered in early January 2022 and consisted of a 2-h remote session enabled by video-conferencing with screen-sharing to demonstrate the D:REACH-HF platform. During the services’ usual triage procedures, newly referred patients were offered participation in the D:REACH-HF study as an option at the time that CR triaging staff offered the REACH-HF programme. Those who wished to take part in D:REACH-HF were allocated to a D:REACH-HF-trained facilitator, who provided the patient with a unique access code for the digital platform.

We assessed feasibility of D:REACH-HF by exploring the following dimensions of feasibility: acceptability (cognitive and emotional responses), appropriateness (perceived fit), adoption (initial uptake/intent), implementation (deliverability/fidelity enablers), practicality/burden (resource and cognitive demands), and maintenance/sustainability (likelihood of continued individual and service use), as articulated in established feasibility and implementation frameworks.^37–40^

We conducted semi-structured interviews with: (1) patients and caregivers after initial use and familiarisation (2–4 weeks into the programme) and towards the end of the programme (10–12 weeks) and (2) facilitators who had been able to deliver D:REACH-HF to a patient during the study period (February 2022 to August 2023). The Interview Topic Guides (Supplemental file) focused on how patients, caregivers, and healthcare professionals (REACH-HF facilitators) perceived and used the D:REACH-HF platform. Interviews were conducted via telephone or video-conferencing by a post-doctoral researcher with experience in interviewing older adults about their conditions and interventions, who was also part of the intervention development team and conducted the think-aloud interviews in stage 1 (RC). The audio-recordings were transcribed verbatim.

A questionnaire pack was sent to patients at week 12 of the intervention to assess the usability of the digital platform (user version of the Mobile Application Rating Scale, uMARS)^41^ demographic data, time since HF diagnosis, and digital device use. D:REACH-HF facilitators were asked to complete a clinical background questionnaire.

#### Data analysis

Sample characteristics and usability ratings were analysed using descriptive statistics (means, standard deviations, and frequency). Interview transcripts were analysed using reflexive thematic analysis,^42^ facilitated by NVivo software (NVivo 12, QSR International Pty Ltd). Inductive and deductive approaches to coding were undertaken. Inductively, the data determined the themes relevant to the aims of this stage. Deductively, the REACH-HF programme theory provided a lens through which we analysed and interpreted data. Two researchers (RC and CT) independently coded interviews for facilitators and barriers to intervention delivery, use, and engagement, and possible impacts from the intervention. The lead author and the researchers discussed patterns in the data, labels, and definitions, and developed and refined the themes, their definitions, and selected potential illustrative quotes and examples. We did not seek data saturation, but aimed for variation in perspectives. After theme development, we organised the qualitative findings at the synthesis stage using established dimensions of feasibility: (a) acceptability, (b) appropriateness (fit), (c) adoption (initial uptake/intent), (d) implementation (deliverability/fidelity enablers), (e) practicality/burden, and (f) maintenance. This mapping complemented, rather than constrained, the thematic presentation and was triangulated with uMARS findings.

#### PPI activities

To enhance trustworthiness and credibility, findings and interpretations were discussed with our PPI group for additional perspectives.

## Results

### Stage 1: intervention planning and design

The resulting guiding principles consisted of the common guiding principles for digital interventions as reported by Yardley and colleagues^27^ and a set of principles specific for D:REACH-HF (Table 2).

Based on content from the paper-based REACH-HF intervention materials and the agreed guiding principles, the draft D:REACH-HF materials comprised a series of digital design screens for three different user interfaces: (1) patient, (2) caregiver (family/friend), and (3) healthcare professional (REACH-HF facilitator).

Each web-based interface included the following screens with:

- a landing page and user log in,
- a main menu depicting the overarching structure and navigation
- example educational content (e.g. excerpts from the original text and imagery from REACH-HF manuals),
- interactive sections involving data entry (e.g. self-monitoring purposes or knowledge testing),
- visual data displays (e.g. display of user-entered data to prompt review of progress and action-planning),
- colour-coded and icon-based calls to action.

The healthcare professional screens also included staff and patient access management (adding, removing).

The web-based interfaces were responsive to computer, tablet, or smartphone screen sizes and included some basic functionality such as clickable buttons for navigation and some functioning data entry sections.

### Stage 2A: intervention optimisation

Findings from the think-aloud interviews centred around four main topics: (1) on-boarding (the process of introducing new users to the platform), (2) navigation, functionality, aesthetics, and accessibility, (3) content of the platform, and (4) facilitation (summarised in Table 3 with more detail provided in the Supplemental file). In some instances, barriers to acceptability and engagement (e.g. negative thoughts prompted by monitoring) are addressed by facilitation from the healthcare professional in the same way as they would be addressed in the original REACH-HF intervention.

**Table 3.** Table of Changes for the D:REACH-HF platform.

Main theme	Sub-themes/key issues identified	Importance indicators	Agreed/proposed change
1. On-boarding	Errors during setup and font clarity issues Desire for clearer password rules and recovery options Option to skip or revisit on-boarding screens	Most participants noted at least one issue	Font size increased; password guidance added; skip and revisit options added
2. Navigation, functionality, and aesthetics and accessibility	*Navigation*: Clear in v2 but missing back buttons and menu overlap *Functionality*: Confusion around icons, labels, and data entry for exercise/medication; mixed views on progress monitoring; desire for clearer graphs and quizzes *Aesthetics and accessibility*: Improvements requested in colour contrast, image quality, and text size; mixed learning preferences (visual, written, interactive)	Reported across multiple participants and PPI reps (EAS, REP, EXP, NCON, IMP, CONT)	Added back buttons; improved icons, labels, and contrast; text re-sizing; mixed-media content (images, quizzes, videos)
3. Content	Wording occasionally perceived as didactic Requests for softer, more person-centred phrasing Positive feedback on empowering tone	Reported by some participants	Wording retained (protected IP) but recommendations noted for future adaptation
4. Facilitation	Importance of facilitator role in setup, tailoring, and ongoing support Need for clear guidance and communication mechanisms	Reported by several participants	To be addressed via facilitator training and external facilitation materials

D:REACH-HF: digitally adapted and enhanced version of Rehabilitation Enablement in Chronic Heart Failure; PPI: patient and public involvement.

#### The D:REACH-HF intervention

The resulting D:REACH-HF programme retained the core components of the paper-based REACH-HF. This includes two progressive exercise programmes (chair-based exercises and a walking programme), stress management (including breathing exercises and relaxation audio), educational materials for both patients (HF manual) and caregivers (Family and Friends Resource), a self-monitoring tool (Progress Tracker), tailored facilitation from a healthcare professional (e.g. HF nurse, CR nurse, Physiotherapist, Exercise Specialist) trained to deliver the programme, and active caregiver involvement in co-facilitating the patient's rehabilitation journey, as well as managing their own well-being.

**Figure 2.**
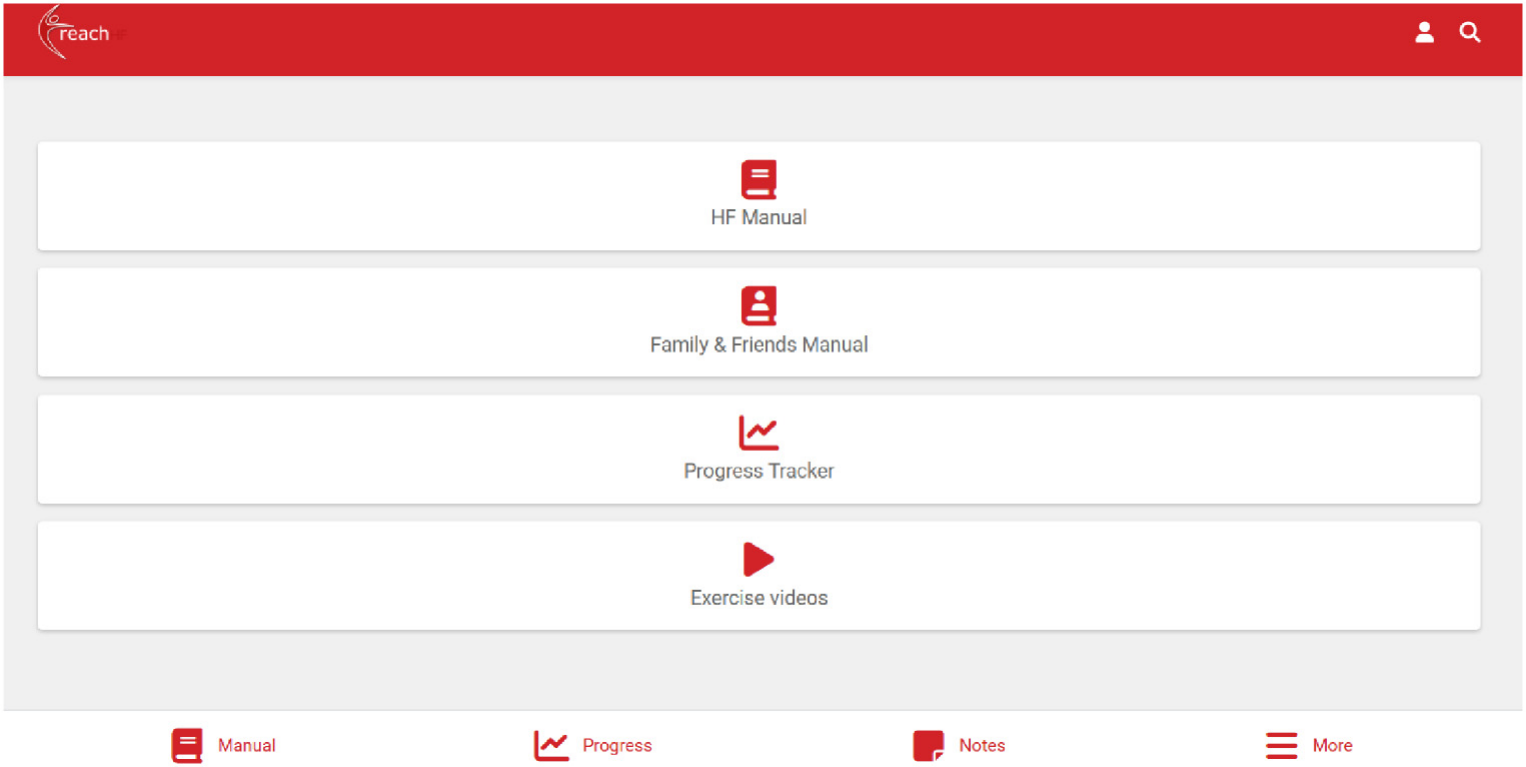
Patient's D:REACH-HF dashboard. D:REACH-HF: digitally adapted and enhanced version of Rehabilitation Enablement in Chronic Heart Failure.

To enable the delivery of these components, the web-based platform (Figure 2; accessible when logging in via: https://reachhf.co.uk) consists of three user interfaces – for patients, caregivers, and healthcare professionals – each tailored to enable self-management, remote support, and guided facilitation.

**Figure 3.**
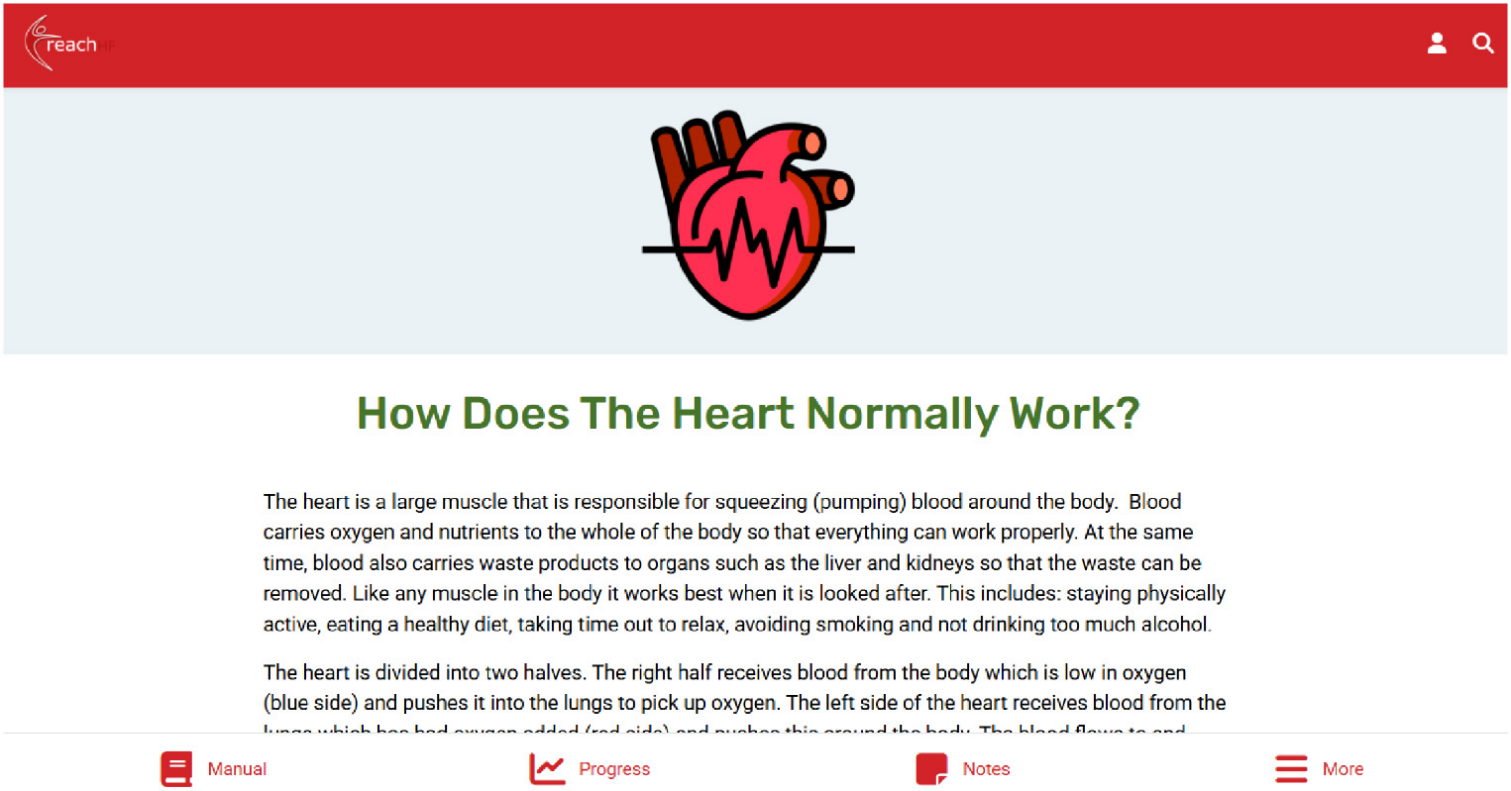
Educational content in the heart failure manual.

**Figure 4.**
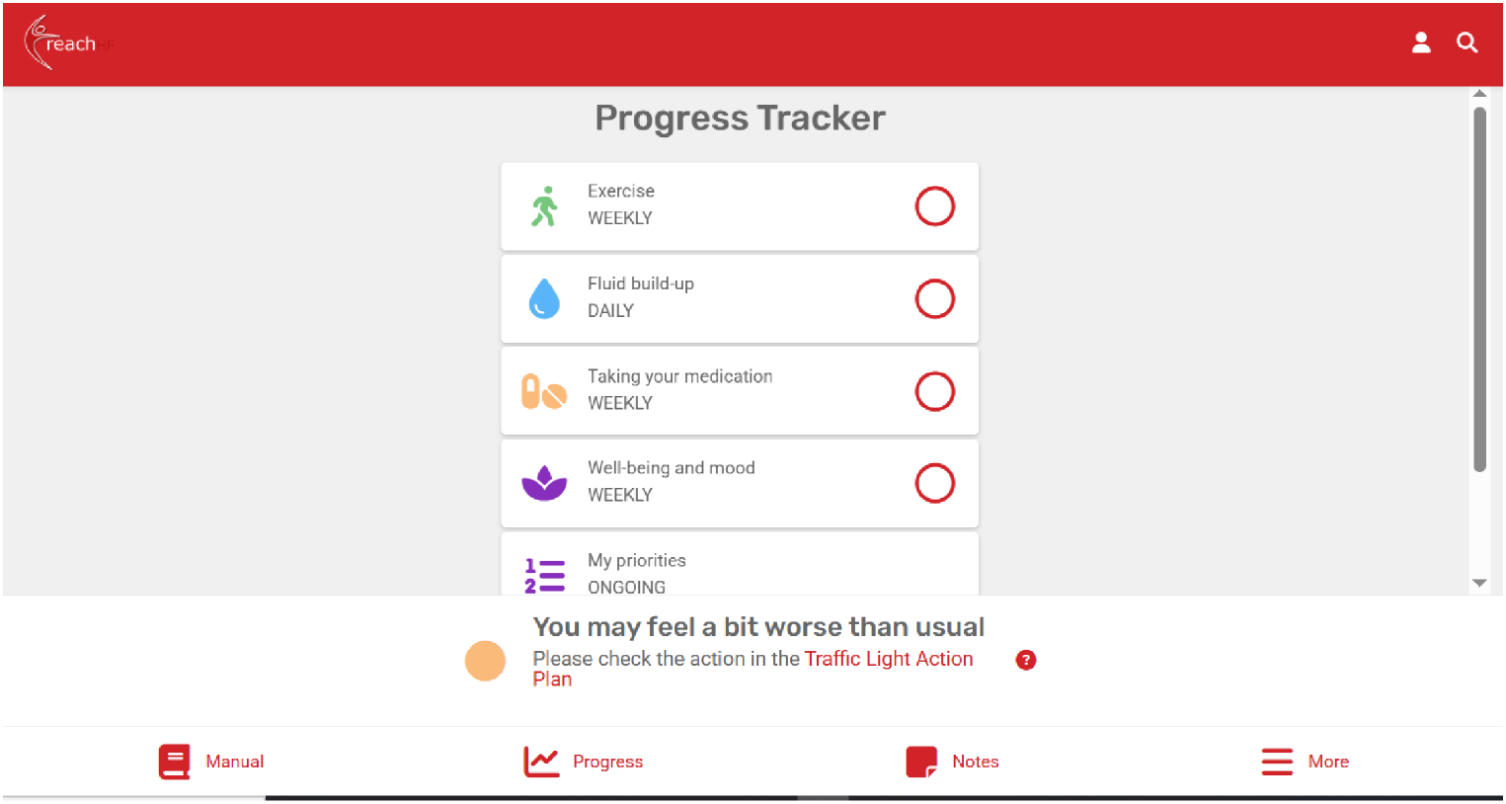
Patient's self-monitoring tool, the Progress Tracker.

Patients can access interactive educational content about living with HF, dealing with uncertainty, and developing skills and confidence for self-management of HF (Figure 3). With facilitation from the healthcare professional, patients set goals for their exercise programme (chair-based, walking, or combined) and their rehabilitation journey more broadly, which they can record in their self-monitoring tool (Figure 4). In this Progress Tracker, patients can enter data on which exercises were conducted and when, and how this felt (exertion). The chair-based exercise videos are accessible on the platform and consist of seven progressive levels. A belly-breathing instructional video is also included.

**Figure 5.**
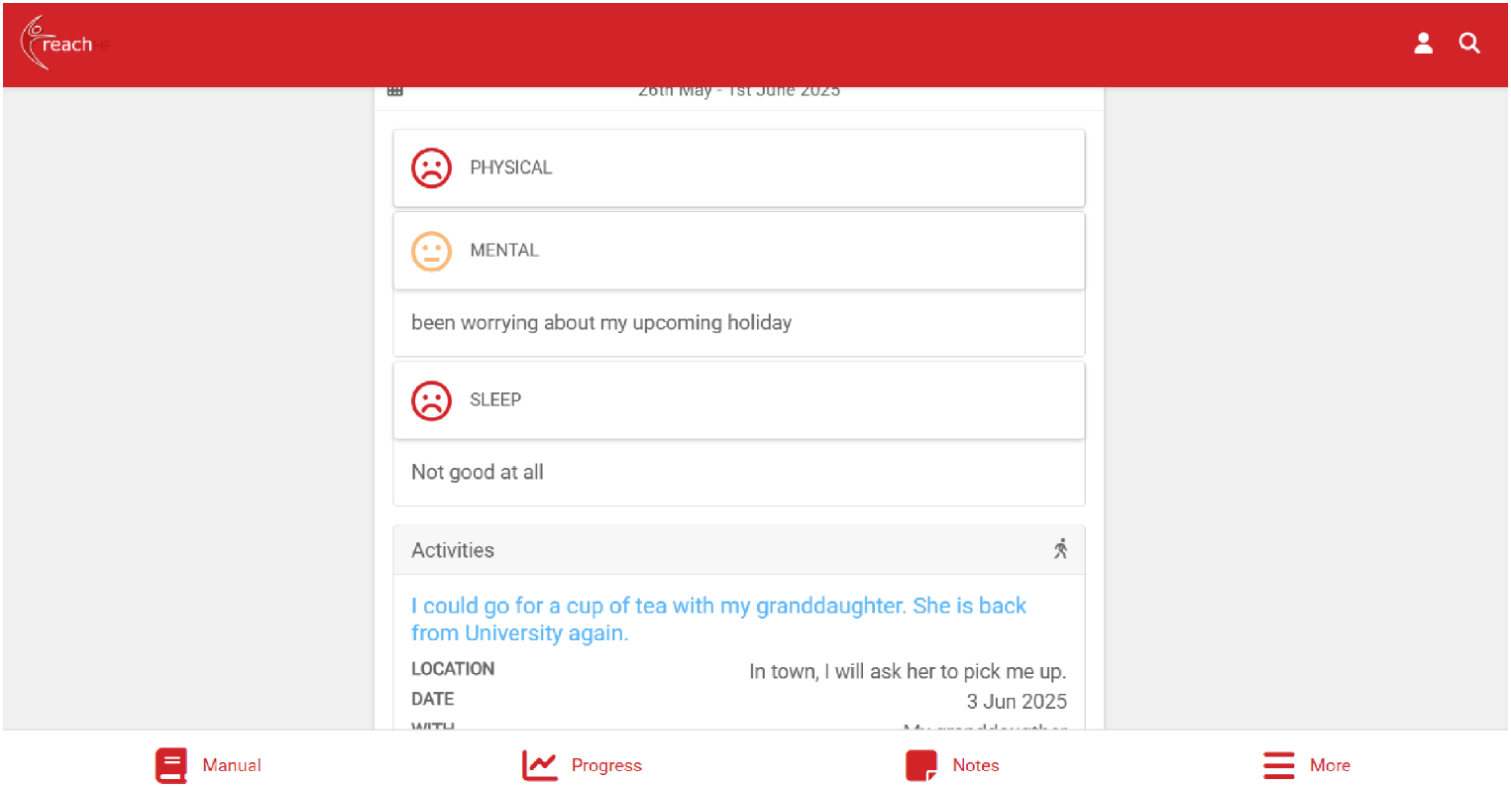
Well-being review and planning.

The Progress Tracker also asks patients to monitor and record symptoms that may be indicators of fluid retention, a key sign of decompensated HF which could result in hospitalisation if not acted upon. These include rapid weight change, ankle and abdominal swelling, sleeping position, and changes in breathlessness, coughing, or wheezing. Patients can also make note of medications, whether they had taken their medications as prescribed, and whether they experienced any side effects. The Progress Tracker also contains a mood and well-being section which focuses on how they are feeling physically and mentally, why this may be, and what they think they could schedule into their goals for the next week to feel better, if needed (Figure 5). As a self-monitoring tool, the Progress Tracker enables patients to reflect on their exercise progress and symptoms to identify what self-management strategies are working for them or not so they can adjust if needed, and when to seek additional support from a healthcare professional. The patient's Progress Tracker data is accessible to their facilitator and any caregiver they wish to share this with.

The caregiver interface enables supportive engagement with the patient's rehabilitation journey (via the Progress Tracker) and offers access to key resources to build their understanding of HF, how it may be impacting their own well-being, how to support the patient in their self-management and how to maintain their own well-being (Family and Friends Resource).

**Figure 6.**
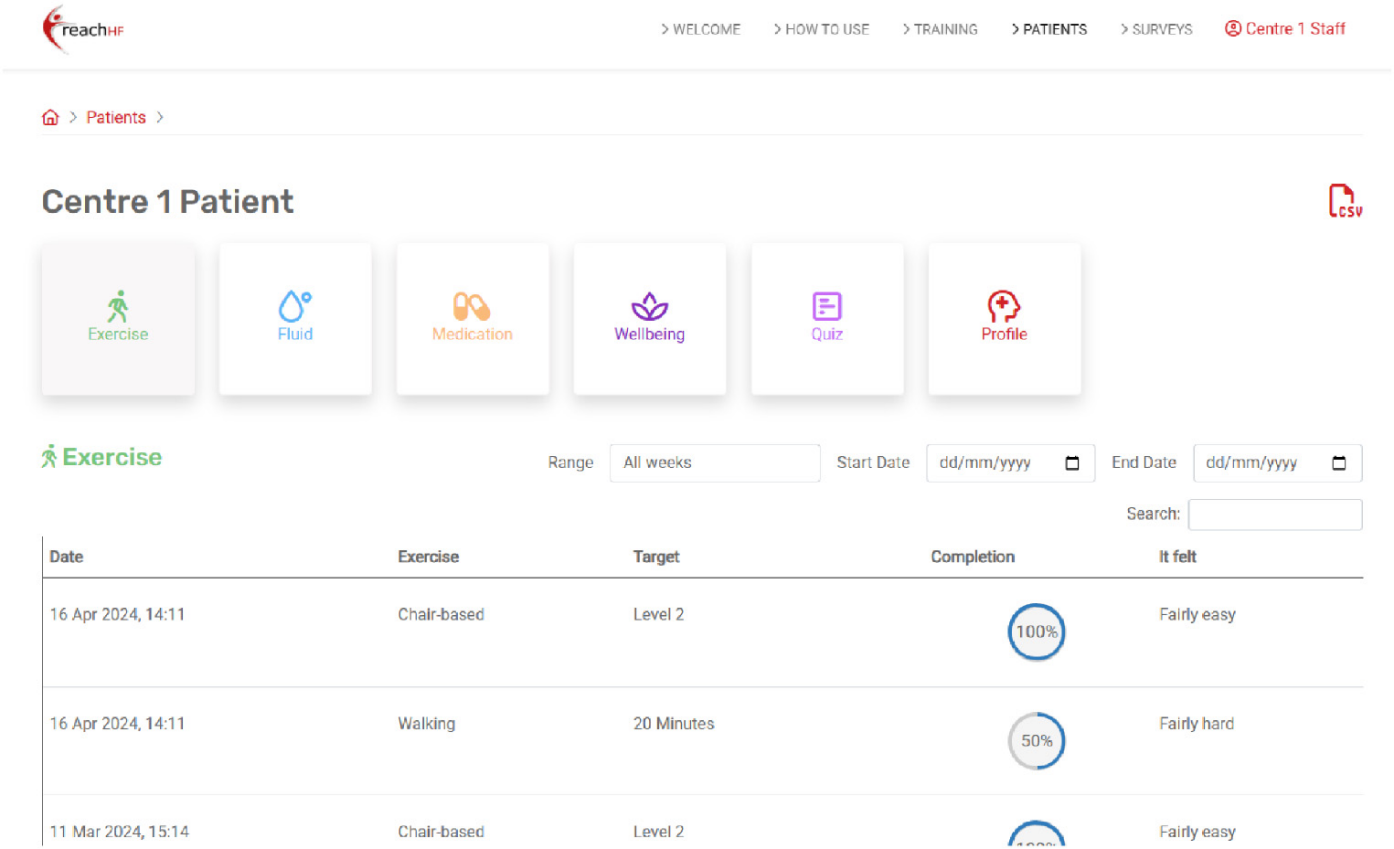
Healthcare professional dashboard – Exercise.

**Figure 7.**
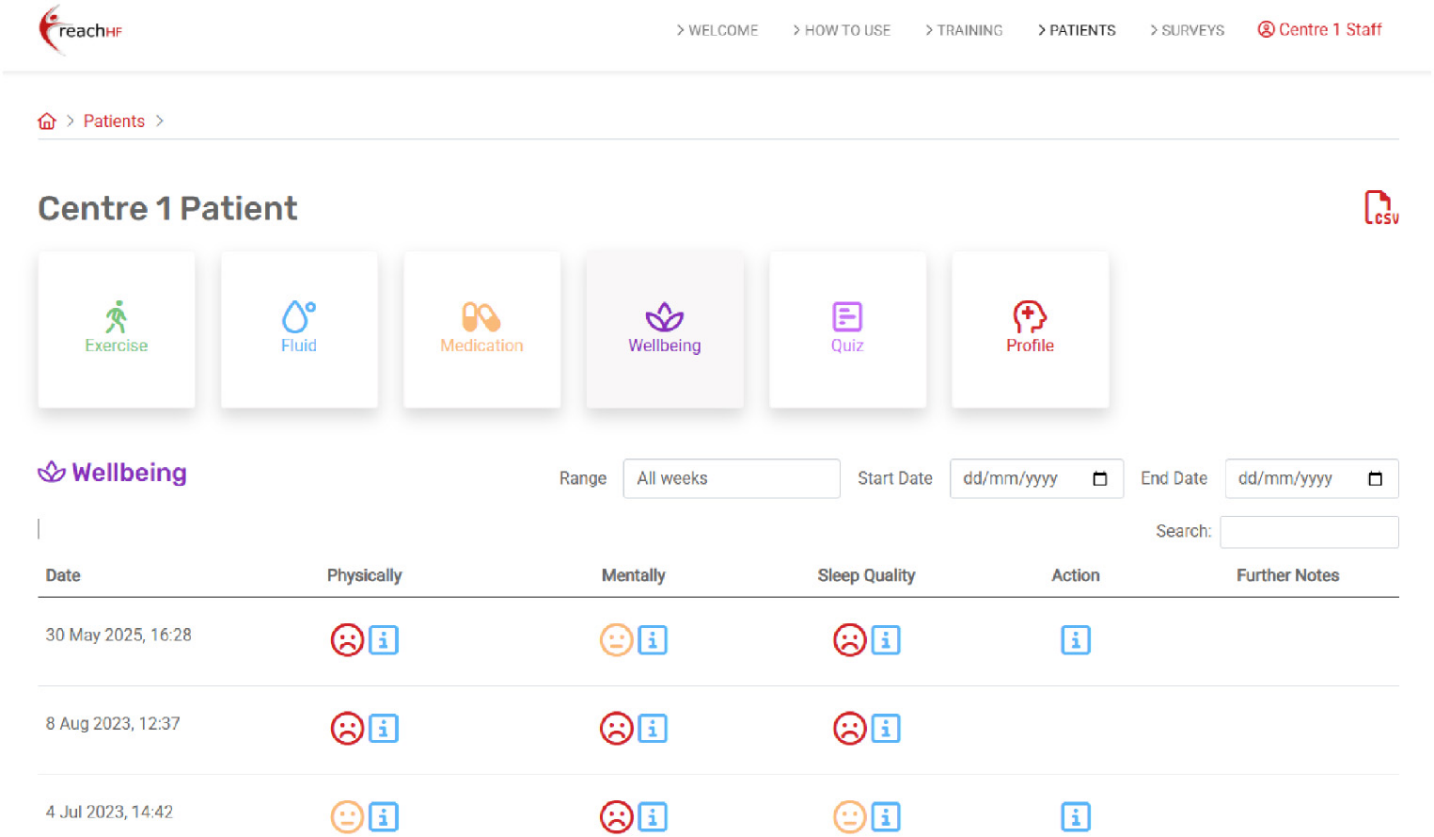
Healthcare professional dashboard – Well-being.

Facilitation, just like the original paper-based REACH-HF programme, is undertaken over a 12-week period by a healthcare professional who has been trained to deliver the programme consisting of approximately two to four face-to-face sessions (at home or in the clinic) and two to four telephone consultations depending on patient needs. A key innovation is the healthcare professional dashboard (Figures 6 and 7) which provides remote access to patient-entered data, allowing facilitators to check patients’ engagement, progress, and needs during the 12-week facilitation period and tailor the support they provide to their patients.

The platform is accessible across multiple devices (smartphones, tablets, and desktops) and features adjustable text sizes and high and low contrast preference settings for accessibility, visual data representations, and intuitive navigation to enhance usability. Integrated facilitation tools, such as the REACH-HF Traffic Light Action Plan for symptom monitoring, and structured communication features such as healthcare professional access to patient's Progress Tracker, ensure that digital delivery remains aligned with the original REACH-HF programme theory.

### Stage 2B: intervention optimisation

#### Participants

**Figure 8.**
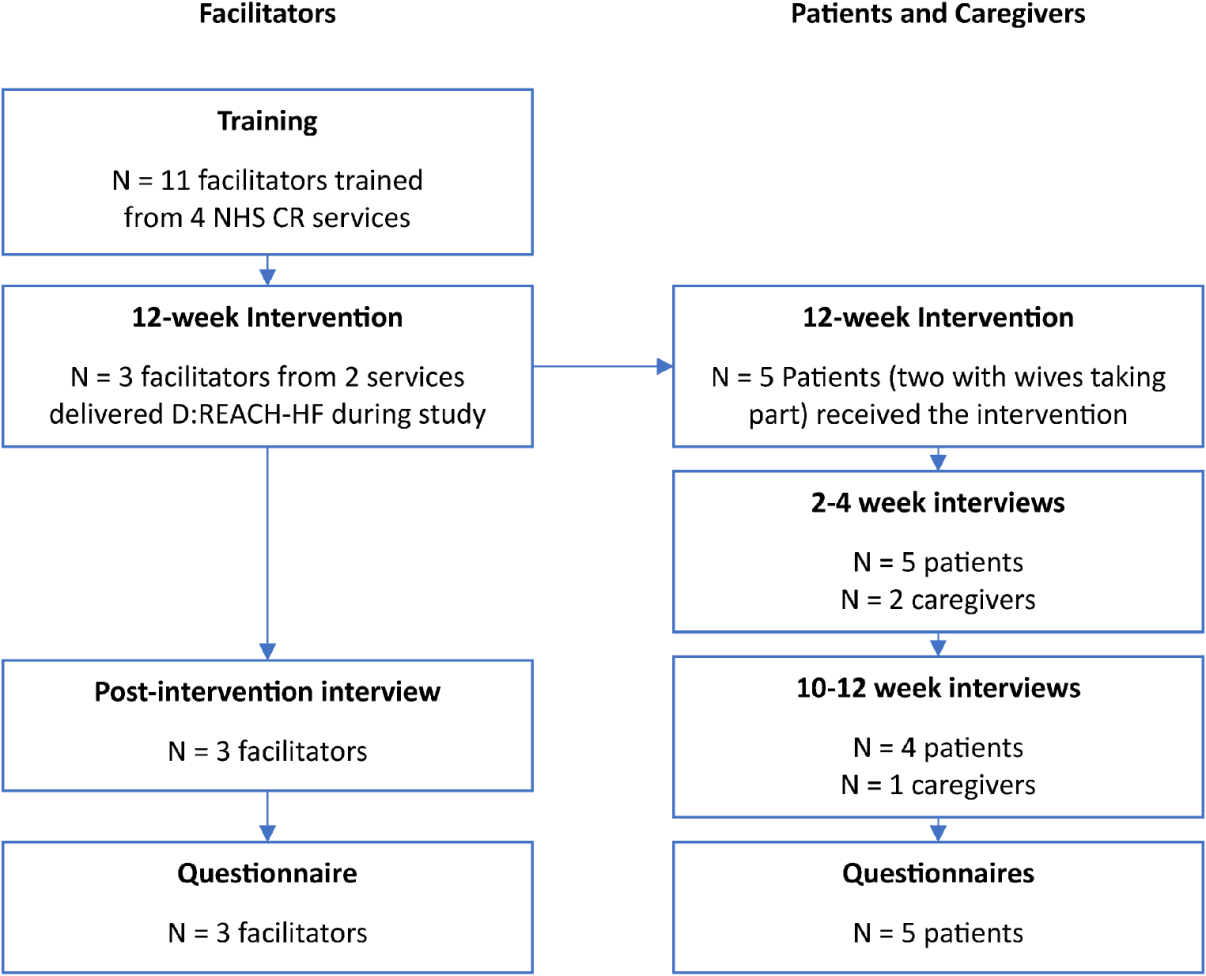
Participant flow diagram.

Three facilitators (all female, Table 4) were able to recruit five patients with HF during the study period (Figure 8). In their questionnaires, they shared previous experience of diverse practices, including in-person and home-based rehabilitation, with adaptations during the COVID-19 pandemic. They highlighted finding the remote verbal assessment of patient engagement challenging. Two facilitators from one site used systems like SystmOne for patient data entry which was often done during consultations, the other reported having gone back to paper-based documentation, although all entered data to the National Audit for Cardiac Rehabilitation. Two facilitators from the same site promoted patient access to data, whereas the other did not. In terms of own device ownership and use, all facilitators owned multiple digital devices and used these frequently for communication, shopping, researching topics of interest, and entertainment.

**Table 4.** Facilitator characteristics.

	Facilitators (*N* = 3)
Mean age in years (SD)	
Female, *n* (%)	3 (100%)
Ethnicity, *n* (%)	
White	2 (66%)
Asian background	1 (33%)
Education, *n* (%)	
Postgraduate degree	2 (66%)
Undergraduate degree	1 (33%)
Experience delivering remote/home CR	3 (100%)
Digital device ownership and use, *n* (%)	
Smartphone	3 (100%)
Tablet	3 (100%)
Laptop	3 (100%)
Desktop personal computer	2 (66%)
Smartwatch	1 (33%)
Use of digital device 5–7 days of the week	3 (100)

CR: cardiac rehabilitation.

All patients were male (see Table 5) and two of these had wives (informal caregivers) who consented to take part. All patients owned multiple devices and were frequent device users. Devices were used for communication purpose (telephoning, emails, and social media), grocery shopping, weather forecasts and news, researching topics of interests, entertainment (music, films, books, and television shows), and photography.

**Table 5.** Patient characteristics.

		Patients with HF (*N* = 5)
Mean age in years (SD)		77 (18)
Male, *n* (%)		5 (100%)
Ethnicity, *n* (%)		
	White	5 (100%)
Education, *n* (%)		
	No qualifications	1
	Higher school certification	1
	Professional qualification	1
	Undergraduate degree	2
Rural vs. urban vs. suburb, *n* (%)		
	Rural	1
	Urban	2
	Suburban	2
Time since diagnosis, *n* (%)		
	0–6 months	3 (60%)
	6–12 months	1 (10%)
	Don’t know	1 (10%)
Type of HF, *n* (%)		
	Reduced ejection fraction	3 (60%)
	Don’t know if *reduced* or *preserved*	2 (40%)
NYHA classification, *n* (%)		
	Class II	1 (10%)
	Class unknown	4 (60%)
Support outside healthcare team, *n* (%)		
	Husband, wife, or partner	4 (100%)
	Ex-wife	1 (100%)
Previously received CR		1 (10%)
Digital device ownership and use, *n* (%)		
	Smartphone	5 (100%)
	Tablet	4 (80%)
	Laptop	4 (80%)
	Desktop personal computer	1 (20%)
	Smartwatch	0
	Use of digital device 5–7 days of the week	5 (100%)

HF: heart failure; CR: cardiac rehabilitation; HFrEF: heart failure with reduced ejection fraction; NYHA: New York Heart Association.

##### Usability questionnaires

Four patients and one facilitator returned their platform usability questionnaires. D:REACH-HF's overall App Quality was high (scoring 4.02; from 1 – Inadequate to 5 – Excellent). Participants gave the highest ratings for Information (quality and credibility) and Functionality (functioning, learning curve, navigation, and flow). Finally, within Aesthetics, lowest ratings were in terms of visual appeal (platform being neither pleasant nor unpleasant). Also in Aesthetics, there were mixed ratings (SD = 1.0) for layout, with the majority considering it to be mostly clear and accessible. Some rated this very low, considering the design to be unclear or difficult to read, locate, or see. Engagement received the lowest score, indicating the need to improve interactivity, customisability, and motivational features (Table 6).

**Table 6.** Usability ratings from the uMARS.

uMARS domain	Mean rating out of 5 (*n* = 5)	SD
Engagement	3.69	0.68
Functionality	4.10	0.52
Aesthetics	3.83	1.09
Information	4.45	0.33
App Quality mean score	**4.02**	**-**

uMARS: user version of the Mobile Application Rating Scale.

#### Qualitative analysis

The themes developed during analysis relate to five key areas: impact of HF and challenges of self-management, use of technology, engagement in CR, facilitation, and perceived impacts. Select illustrative quotes are presented here. All quotes are edited to remove some repetitions and ‘errs’ and ‘umms’ and reported with pseudonyms for anonymisation while maintaining the human element.

##### Impact of HF

Participants described the profound impact HF on their physical and emotional well-being, as well as the challenges they faced in self-managing their condition before joining D:REACH-HF. This was corroborated by facilitator experiences of managing patients with HF.

###### Physical and emotional toll

Participants described how HF forced them to alter their activities and hobbies, leading to a loss of independence. One patient reflected on their progressive decline in physical ability: ‘We thought well, we can’t do what we’d like to do’ (Patrick, patient, 2–4 weeks). These limitations also created significant emotional strain, as participants grappled with the loss of activity levels and independence expressing feelings of frustration, helplessness, and even depression.

I mean I realised that I had got quite depressed, I suppose looking back at it now. Where, because of my reducing exercise, and capacity for it (Patrick, patient, 2–4 weeks).

###### Self-management challenges

The physical and emotional struggles were compounded by challenges in self-management. Many patients relied on their own intuition for self-management, often without structured guidance which resulted in misunderstood or overlooked key symptoms, or did not know that that self-management was a necessary part of their care because ‘I wasn’t aware of having to manage it beforehand’ (Matthew, patient, 2–4 weeks). Only one caregiver mentioned a patient booklet having been received upon her husband's discharge from hospital which they had been following.

I didn’t realise that this was so much fluid retention. I knew that I was getting bloated…from time to time, but put it down to indigestion type of thing (Patrick, patient, 2–4 weeks).

##### Use of technology

The use of technology as a way of delivery was thought to increase access to rehabilitation and highlighted that ease of use is important for ongoing engagement.

Once things start getting a bit complicated [with any technology], it's… it makes you less and less want to use it. But no, it's [D:REACH-HF] simple and to the point. And it's that, that makes you use it (David, patient, 2–4 weeks).

Although all participants owned and frequently used multiple digital devices (see Tables 4 5), participants reported varying levels of *digital competence*, affecting their delivery of, and engagement with, the D:REACH-HF platform. While some were comfortable using digital tools, others faced challenges due to limited familiarity, vision impairments, or reliance on caregivers for access.

My wife is doing the – is doing the whizz keying on the board and I just fall in with you know what I’m instructed – what I’m supposed to do (Matthew, patient, 10–12 weeks).

Logging in and navigating the platform posed difficulties for several users, with issues such as password failures, not being able to find the password recovery emails, unclear instructions, and perceived limited accessibility on different devices (e.g. preference for iPads over computers). *Technical issues* significantly impacted engagement, with users struggling to backdate information, input data easily (e.g. lack of confirmatory messages), and understand certain platform features (e.g. traffic light indicators) which may create uncertainty about engagement in subsequent self-management behaviours.

I didn’t really understand whether we were supposed to say we were on ‘red alert’ – we were worried? Or, we’re green – we’re absolutely fine? Or, whether that would come up as a traffic light, based on the information we’d given? (Ann, caregiver, 10–12 weeks).

Despite these challenges, patients reported that facilitators were instrumental in overcoming initial difficulties, offering tailored guidance to resolve issues, navigate the platform, and ensure they could effectively track their progress. However, one participant who had received the paper-based manual due to initial struggles in accessing his D:REACH-HF account, found the digital platform less intuitive compared to the paper manual, leading to frustration and disengagement.

While patients encountered varying degrees of difficulty with the platform, facilitators highlighted how they supported engagement by troubleshooting issues and providing guidance. However, one facilitator, despite their digital expertise, stressed that it had been challenging to recruit patients for D:REACH-HF, suggesting that their patient base may not have been the ideal target population for digitally-supported CR delivery. However, a facilitator at another site reported that her own beliefs about a possible target audience had gotten in the way of offering REACH-HF to some who may well have benefitted from it.

Actually, if I looked on paper, before they all came in, I probably would have ruled a few of them out, who actually are on it now, because I thought to myself… especially the patient who's ninety! I did think oh, he's probably not wanting to be bothered with putting stuff in a laptop before I even met him! But I was only like five or ten minutes into the conversation and I was chatting to him and I thought oh, and then I asked him what he did for a living and he said. So, I said, do you do a lot on your laptop and a lot of stuff like that? He said, oh, a lot, yeah. He said, yeah, because that was part of my job. So, when I explained it [D:REACH-HF], he said, oh, that's right up…have you got anything I could do like that? He even asked me! So, yeah. So, I think you never know until you have a good chat with the patient (Fiona, healthcare professional).

##### Home-based CR

###### Expectations

Many participants expressed initial uncertainty about what CR involved or what to expect, with some reporting that they were unaware of available support, until they actively sought it out. Expectations of CR ranged from hopes of structured guidance and support in regaining physical function to assistance in understanding their condition better. Some participants anticipated clear direction on exercise, symptom monitoring, and lifestyle adjustments, while others saw it as a way to stay connected to healthcare professionals and ensure they were on the right track.

I want to improve my health. I know it will never come back or go back to what it was. But as long as I can stay fairly mobile and still be a carer to my wife (Matthew, patient, 2–4 weeks).

###### Accessibility

As a home-based programme, D:REACH-HF offered participants the flexibility to engage with rehabilitation on their own terms, which was seen as both an advantage and a challenge. Participants appreciated that ‘If things pop up you go in and look for it. Like, you can find an answer, or a solution, which puts me back at ease. But it's all there as well. It's there for when you need the help, or suggestions when you need the support and it makes things easier to get done’ (David, patient, 10–12 weeks), the ability to integrate exercises and self-monitoring into their daily routines, and the ability to go at their own pace.

It's something that I want to get into not that fast, but slowly [HF manual]… I am getting lots of information out of it…Digesting the manual bit by bit…Little and often (David, patient, 2–4 weeks).

###### Self-monitoring

Participants increasingly engaged in self-monitoring of exercise, symptoms, and well-being as they became more familiar with the digital data entry process, transitioning from initial confusion to regular use. For some, tracking progress served as a motivator, encouraging them to stay active and maintain their self-management behaviours.

Yes, I think if you look at it, we’ve actually – we’ve got better! So, we were inputting very little data at the beginning, because it was confusing us. But we’re now – we’re now doing it most days (Helen, caregiver, 2–4 weeks).

###### Supporter engagement

Participants engaged family and friends in their rehabilitation, fostering both physical activity and social well-being. Caregivers played a crucial role in motivating patients, reinforcing the benefits of exercise, and supporting adherence to routines. However, there were some concerns about overburdening the caregiver.

…well, she could [get involved] but …she is waiting for knee replacements…she's got so much on her plate.…I wouldn’t want, you know, for her to have more responsibility (Patrick, patient, 2–4 weeks).

Some participants exercised alongside family members, such as daily walks with loved ones or completing the chair-based exercises together. Others incorporated social outings, like coffee trips. Family members also helped with self-monitoring and tracking progress, providing emotional support that contributed to increased confidence and motivation.

However, during the programme, some caregivers also learned that providing too much support may be detrimental to the patient's ability to retain functional capability for activities of daily living.

Like, we had a discussion about I would take his breakfast up for him and make sure he had his tablets on time, just in case he’d slept through. And then we realised that I was like doing things for him, that he should be doing himself. So, we decided that he would come down and have his breakfast. So, it could have been in that period where I was perhaps over-caring because of my worries (Helen, caregiver, 2–4 weeks).

###### Barriers to engagement

Participants faced several barriers to engaging with their CR programme, aside from the technological issues reported above. Many of the barriers related to health challenges (including those of the caregiver), motivation, and competing responsibilities. For some, ongoing medical conditions, post-surgical recovery, and other co-morbidities, made it difficult to fully participate in or prioritise HF rehabilitation.

One of the difficulties was that he's had so many different things going on. He's had a heart operation. Then he had a stroke. I don’t know if you know that? Two weeks after he came out (Helen, caregiver, 2–4 weeks).

Additionally, participants with low mood found it harder to maintain motivation, particularly in the early weeks of the programme. Caregivers also mentioned difficulties in balancing their loved one's rehabilitation with their own work and family responsibilities, making it harder to prioritise the programme. Most participants described environmental factors, such as poor weather, which temporarily limited their ability to engage in the walking programme. Despite these barriers, many patients found ways to adapt, often with support from family members or facilitators, gradually integrating the programme into their daily routines.

##### Facilitation

While digital tools were seen as potentially useful, most emphasised that human support remained essential and could not be replaced by technology. The role of facilitators was central to participant engagement with their CR journey. They provided technical support, guided patients, and caregivers in navigating the platform, interpreted progress, and adapted the programme to individual needs. Beyond technical guidance, facilitators played a motivational and emotional support role, offering encouragement, reassurance, and accountability through regular telephone check-ins. Many patients and caregivers reported that the human connection offered by facilitators made the programme feel more engaging, reduced feelings of isolation, and reinforced their commitment to self-management.

So, the support is REALLY great! It's very uplifting and very positive, and [facilitator] has rung me up a couple of times to ask after like what's happened like, if I’ve had an appointment, or anything like that. Yes, so, I’m not left to dry, as they say! From a mental point of view, it's very reassuring (David, patient, 2–4 weeks).

The knowledge that someone was able to see their self-monitoring data remotely provided a *sense of oversight*, making the process feel more meaningful and reducing feelings of isolation in managing their condition. Participants appreciated that facilitators could identify potential health concerns and intervene when necessary, adding a layer of security to the home-based approach. This was not only clear from patient and caregiver accounts, but the increased patient motivation through this oversight also became clear from facilitator accounts.

What they’re telling me is they’re really motivated because they're logging it on there and they know that we can see it and they know that we're going to be ringing them with what we, with what they've logged on there as well (Rose, healthcare professional).

###### Facilitator perspective on digitally-supported CR

Most facilitators reflected on how care delivery had changed during and since the COVID-19 pandemic. Telephone consultations had been embedded more and more as normal practice. However, these were seen as time-consuming, and ineffective for assessing engagement with CR and personal tailoring of care due to relying on retrospective accounts from patients. D:REACH-HF seemed to overcome those concerns about telephone consultations, by allowing participant to log their progress daily rather than waiting for their consultation and having to remember everything they had done.

We have been doing a lot of calls. And, they’re very time-consuming, calls! And, they don’t always give you what you need. I’m not a fan of doing lots of calls, because sometimes you don’t see the patient. I know you don’t see them when you’re doing this [D:REACH-HF], but they can tell you anything…. So, I felt as if what you were viewing was the real thing. They weren’t sort of giving you any flannel as well! You often get that with patients, who just tell you what you want to hear (Carolina, health professional).

Facilitators generally viewed D:REACH-HF as an effective tool for improving rehabilitation delivery and reported that most of their patients were eager to use the platform.

Most of the patients, when I got there had already opened it all up. They’d been on and had a nosey and they’d seen it anyway (Fiona, healthcare professional).

One of the most significant advantages of D:REACH-HF reported by the facilitators was that it enabled remote access to patient progress data. This feature allowed facilitators to prepare for consultations more effectively, focus on key concerns, and tailor support accordingly, making interactions more efficient and personalised.

I found that so helpful to be able to have all of that information before you've called them and you can kind of have a plan in mind of what you want to do before you ring them. And I just think it makes the conversation easier as well because they're not having to remember everything because you've got it in front of you while your speaking to them (Rose, healthcare professional).

This feature was also considered ‘a good measure, basically to see how involved a patient is in trying to do, you know, in his exercises and trying to do the rehab side of the heart failure’ (Carolina, healthcare professional). Moreover, facilitators reported that the digital platform encouraged patient autonomy, as individuals were more likely to self-monitor their symptoms and remain engaged in rehabilitation.

The fact that I never thought he’d move up any levels. But he has! And, he took ownership. And it was good to see that. And, I said to him who knew, you know, that this would happen? And, he said: yeah. He goes: I’m surprised myself! And he says: and I’m just…I’m like a new man! So, that might not have been just because of that. But it may have been just to the fact that, you know, he was putting stuff in and he knew somebody was going to read that and was going to look at that properly and sort of acknowledge it (Carolina, healthcare professional).

###### Patient requests for long-term access

A key finding from facilitator interviews was that all participating patients expressed a strong desire for continued access to D:REACH-HF beyond the study period. Facilitators noted that patients valued the platform as an ongoing self-management tool, particularly for tracking symptoms, maintaining exercise routines, and being able to access HF education when required in the future. One facilitator highlighted that patients viewed D:REACH-HF as something they could keep coming back to, suggesting its potential role in long-term condition management. However, it was also highlighted that patients would need clarity that there would be no healthcare professional accessing their data (no oversight) beyond the 12-week facilitated programme.

Every one of these – of my patients has asked that question. That was the first thing they said. Can we keep this platform? Can we still have access to it? And, they all know they’re not going to get a call. They all know that. But they are desperate to keep it (Fiona, healthcare professional).

##### Perceived impacts from D:REACH-HF

By engaging with the educational materials and self-monitoring tools and support received from their facilitators, participants gained a clearer picture of their condition. This knowledge helped to reduce uncertainty and fostered a sense of control, making them more proactive in managing their symptoms and treatment. For some, having structured guidance from the programme and facilitator reinforced their ability to take ownership of their health, helping them navigate daily life with greater confidence.

… now, with this programme, it's given me a more optimistic outlook (Matthew, patient, 2–4 weeks).

As patients and caregivers became more comfortable with self-monitoring and tracking their health data, they also reported an increase in self-management behaviours. Many started engaging more consistently in activities such as recording their weight for fluid retention, monitoring symptoms like breathlessness and swelling, and staying active to identify and prevent worsening health.

I check it every day, because my legs, my ankles and my legs look good. No swelling. But you delude yourself. So, I check my weight every day. Make sure it's alright. That's what the whole programme makes you do. It makes you think about what you’re up to and what you’re doing. It's – yeah, it's, you know, become part of my routines – exercise routines that is and how I’m feeling and all the rest! But I think that's what it's supposed to do. To make you think. If you’re just blasé about it, you’re just going to miss everything (David, patient, 10–12 weeks).

The programme provided structure and motivation, encouraging participants to integrate these behaviours into their daily routines. For some, the act of self-monitoring acted as a motivator, pushing them to be more mindful of their exercise levels and overall well-being.

… in reviewing progress and so on, it's acted as an incentive really to do exercise and keep an eye on my weight, and that type of thing, been an incentive to sometimes get off my backside and do something! (Patrick, patient, 10–12 weeks).

These changes translated into noticeable health and well-being benefits, which in turn motivated continued engagement in the programme and sustained self-management among other competing priorities.

Other people may not have had so many distractions as we’ve had. But we’ve been convinced of its usefulness, which is why we’ve kept with it (Helen, caregiver, 10–12 weeks).

Some participants experienced improved exercise capacity, as they were able to increase their physical activity levels gradually. Others reported feeling more positive and in better spirits, as the programme gave them a sense of progress and achievement. The ability to track and reflect on their improvements helped reinforce these feelings, reduced anxiety, and improved overall emotional well-being.

He's… had days where he's really seen the difference. Where he's walked more strongly. Or, walked more quickly. So, he can see the benefit of doing the exercises too – to being able to walk better (Helen, caregiver, 2–4 weeks).

In some cases, the programme helped shift relationships, with participants taking more responsibility for their health rather than relying entirely on family members. This sense of independence was beneficial both for the individual and their loved ones, fostering a shared approach to managing HF.

…at the beginning we did them [exercises] together… And then he's become more independent. So, he does them by himself (Helen, caregiver, 10–12 weeks).

In summary, patients and caregivers engaged with the programme hoping for physical and mental health improvements, while facilitators aimed to improve accessibility to CR by providing another alternative to centre-based programme. Continued engagement with the programme was motivated by: (a) patient enjoyment of self-monitoring exercise progress, medication, weight, and well-being, (b) structured guidance and oversight from facilitators, and (c) perceived improvements in health and independence resulting from the programme. Facilitators reported that remote access to patient data enhanced their ability to personally tailor the delivery of the programme by enabling more efficient patient-centred follow-up consultations. The flexibility of D:REACH-HF allowed patients to participate in CR from their own home, integrate their rehabilitation into their day-to-day routines, and go at their own pace, addressing key limitations of traditional centre-based CR. Participants’ engagement with D:REACH-HF was shaped by their digital competence, social support, and the platform's usability and flexibility. Despite overall positive feedback, some barriers to engagement included technical difficulties, missing functionalities, and digital competence challenges, though these were often overcome with facilitator and caregiver support. Health-related setbacks also affected participation, but the programme's adaptability helped patients resume CR when feasible.

### Synthesis

To situate the qualitative findings within the dimensions of feasibility, Table 7 summarises stage 2B results across: (a) acceptability, (b) appropriateness (fit), (c) adoption (initial uptake/intent), (d) implementation (deliverability/fidelity enablers), (e) practicality/burden, and (f) maintenance/sustainability.

**Table 7.** Stage 2B findings mapped against key dimensions of feasibility.

Domain	Summary of stage 2B findings	Supporting signals from stage 2B
Acceptability	D:REACH-HF was well-received; participants valued structured guidance plus human facilitation; strong positive sentiment towards the platform.	**uMARS app quality** **=** **4.02/5** (*n* = 5); highest sub-scale **Information** **=** **4.45**. Universal patient **requests for continued access** beyond 12 weeks.
Appropriateness (fit)	Home-based delivery fit daily routines (‘bits and pieces’), reduced travel burden, and felt reassuring due to facilitator visibility of self-monitoring data; caregiver involvement generally aligned with household roles (with attention to avoiding over-caring).	Qualitative reports of flexible pacing and meaningfulness of logging due to remote oversight.
Adoption (initial uptake/intent)	Patients engaged despite varied digital competence, often **increasing use over time. ** Some site-level **recruitment challenges.** Strong **intent to continue** use.	Interview accounts (e.g. moving to ‘most days’ use); facilitators noting challenged assumptions about older adults’ digital use.
Implementation (deliverability/fidelity-enablers)	Clinician dashboard **enabled efficient tailoring** and focused consultations. Human facilitation remained **essential** (tech support, motivation, interpretation). Implementation pain points: **on-boarding/password recovery**, **icon/label/traffic-light clarity**, **backdating entries**, **font/contrast**, and **device preferences**.	**uMARS functionality** **=** **4.10**; interview evidence of dashboard use to prepare calls and personalise support.
Practicality/burden	Engagement balanced against **co-morbidities**, **low mood**, **weather**, and **competing responsibilities.** Caregiver burden surfaced but was **manageable** through adjusted roles. Flexibility supported pause-and-resume.	**uMARS aesthetics** **=** **3.83** (mixed layout/visual appeal); lived-experience accounts of adapting routines.
Maintenance/sustainability	**Patients:** universal desire to retain access post-facilitation for ongoing self-management. **Facilitators:** viewed continued service use as feasible and valuable (efficiency and personalisation from remote data review), contingent on addressing on-boarding/UX pain points, integrating with local workflows/documentation, offering it consistently to eligible patients, and clearly communicating lack of clinician oversight beyond 12 weeks.	Universal patient requests to keep access; facilitator accounts of dashboard-enabled efficiency/personalised follow-up; noted recruitment variability at one site; reported on-boarding/password recovery and icon/traffic-light clarity issues; device preferences; explicit need to clarify no ongoing clinical monitoring after 12 weeks.

D:REACH-HF: digitally adapted and enhanced version of Rehabilitation Enablement in Chronic Heart Failure; uMARS: user version of the Mobile Application Rating Scale; UX: user experience.

Overall, signals for acceptability and appropriateness were strong, with patients engaging at home and valuing blended digital–human support. Implementation was feasible and clinically useful (dashboard-enabled tailoring) but exposed specific user experience (UX)/on-boarding targets and accessibility tweaks to optimise engagement. For maintenance, patients expressed universal intent to continue use, while facilitators indicated service-level continuation was feasible, conditional on workflow integration, consistent offering, and clear boundaries after the 12-week facilitation.

## Discussion

This study co-developed D:REACH-HF, a digitally-enhanced, NHS-ready version of the clinically effective^18^ REACH-HF home-based CR programme for HF. Interviews with patients, caregivers, and healthcare professionals (REACH-HF facilitators) indicated that D:REACH-HF was well-received, and this was supported by high app-quality scores. Engagement was the lowest-rated usability domain on the usability questionnaire, highlighting opportunities for improving interactivity and motivational features. Importantly, all patients requested continued access to D:REACH-HF, despite knowing there would be no ongoing external oversight and facilitation, indicating its perceived long-term value for self-management and rehabilitation. Our higher-level synthesis indicated strong acceptability and fit, with feasible implementation that highlighted specific on-boarding and UX improvements to raise engagement. Maintenance prospects were positive, as patients intended to continue use, but facilitators judged feasibility to depend on workflow integration and clear post-facilitation boundaries.

Positive attitudes of patients and caregivers towards digital health technologies were consistent with existing research^43–45^ and reflect broader trends in increased digital adoption among older adults.^46^ Digital competence varied, with some participants facing initial challenges. Caregiver and facilitator support helped overcome these barriers and echoes evidence that social support, including healthcare professional facilitation, underpins engagement among individuals with lower digital literacy.^47–50^ The ability of participants in D:REACH-HF to navigate these challenges is particularly relevant given the established link between sustained digital engagement and long-term adherence to rehabilitation programmes, and associated clinical benefits.^47^ Participants generally found the platform intuitive and adaptable to daily life. That matters because continued use of home telehealth services in HF and chronic obstructive pulmonary disease (COPD) depends on seamless integration into routines and a positive UX.^51,52^ Older adults with chronic conditions are more likely to engage when digital interventions are minimally disruptive,^53^ which aligns with our finding that D:REACH-H supported engagement despite technical challenges. Given that technology acceptance is an evolving process shaped by initial attitudes and ongoing support from caregivers and healthcare professionals,^47^ facilitators were particularly important early on for on-boarding and troubleshooting.

How participants processed information also played a critical role in their engagement with D:REACH-HF. Prior research suggests that information overload can discourage engagement and hinder behavioural change.^54,55^ Consistent with this, patients and caregivers in our study reported that the flexibility of D:REACH-HF enabled them to engage with the educational content in ‘bits and pieces’, allowing them to absorb information gradually at their own pace, without feeling overwhelmed. Additionally, participants emphasised the REACH-HF facilitator as a key source of reassurance, support, and guidance; this reflects existing evidence that digital technology is most effective when it complements, rather than replaces, human facilitation.^51,56^ The facilitator's role was particularly valuable in sustaining engagement by providing structured guidance and addressing uncertainties, underscoring the importance of blended digital–human support in remote CR programmes.

The benefits of D:REACH-HF align with and build upon findings from previous evaluations of REACH-HF.^21–23^ D:REACH-HF seems to retain the core benefits of REACH-HF, such as increasing understanding of the condition and increasing skill and confidence in self-management, while leveraging digital technology to enhance remote facilitation and support engagement in self-monitoring behaviours. While D:REACH-HF is equally flexible as the paper-based REACH-HF version, a key distinction is that healthcare professionals have remote access to patient self-monitoring data, including exercise progress, medication adherence, fluid retention, breathlessness, and overall well-being. This real-time visibility during the facilitated period of the programme enhances perceived safety and security for patients, as they feel a sense of oversight in their rehabilitation journey.

Remote access to patient data was perceived to optimise the efficiency of personalised care. Rather than spending consultation time gathering routine progress updates, facilitators can use this data to tailor their guidance and interventions more precisely. These efficiencies may help to mitigate some of the barriers to implementation of home-based CR for HF that were identified in the REACH-HF NHS Beacon Site study (i.e. resource constraints and staff engagement).^23^

### Strengths and limitations

A key strength of this study is that we have digitally adapted an existing and effective CR programme for HF that has already been adopted and is being delivered in the NHS. By building on an established intervention, D:REACH-HF leverages evidence-based principles of self-management and rehabilitation,^12^ while enhancing accessibility and optimising engagement through digital delivery. Another strength of this study was the use of the Person-Based Approach^26^; this provided a structured framework for intervention development, ensuring that our target audience's perspectives were integrated throughout the design and optimisation process. Moreover, our co-development approach included actively involving patients with HF, their caregivers, and healthcare professionals involved in the delivery of CR to shape and refine both our research and D:REACH-HF. Few Person-Based Approach projects report involvement of their target population in this way alongside their qualitative research participants.^57^ This combined method enhances the likelihood of long-term acceptability, feasibility, and ultimately effectiveness in real-world implementation.^26^

However, this study has several limitations that need to be acknowledged. Firstly, we experienced difficulties in implementing the study during the COVID-19 pandemic (during which many NHS CR staff were redeployed or not working due to illness). This resulted in delayed study set-up and recruitment of patient and caregivers receiving D:REACH-HF. Our PPI and research participant groups for the different stages were limited in socio-economic and ethnic diversity. To address inequities going forward, further optimisation of D:REACH-HF to ensure it meets the needs of people from diverse backgrounds in terms of relevance and accessibility is needed.

## Conclusions

This study reported the development of D:REACH-HF, a co-developed, digitally-enhanced, NHS-ready version of REACH-HF, and demonstrated the feasibility and acceptability of supporting self-management and engagement in home-based CR for HF. By leveraging digital technology, D:REACH-HF retained the core benefits of the original programme while ensuring accessibility and usability, and enhancing facilitator oversight through remote patient monitoring. Self-monitoring, structured facilitator support, and the flexibility of digital CR were central to patient engagement. Barriers remained, including technical challenges and variable digital literacy. In addition, limited diversity in participant representation is a limitation that constrains transferability and underscores the need for inclusive optimisation and targeted implementation strategies. Future research should evaluate uptake, sustained engagement, and clinical outcomes, and ensure equitable access to digital CR. As health systems adopt digital health interventions, addressing implementation barriers, strengthening digital inclusion, and refining hybrid models will be essential to maximise impact and reach.

## Supplemental Material

sj-docx-1-dhj-10.1177_20552076251406545 - Supplemental material for Digital adaptation of the clinically effective REACH-HF home-based cardiac rehabilitation programme for people living with heart failure (D:REACH-HF) Supplemental material, sj-docx-1-dhj-10.1177_20552076251406545 for Digital adaptation of the clinically effective REACH-HF home-based cardiac rehabilitation programme for people living with heart failure (D:REACH-HF) by Samantha B van Beurden, Rosina Cross, Sinéad T J McDonagh, Liz Clark, Chloe Thomas, Colin J Greaves, Patrick Doherty, Rod S Taylor and Hasnain M Dalal in DIGITAL HEALTH
