# Supplementary material for: Digital adaptation of the clinically effective REACH-HF home-based cardiac rehabilitation programme for people living with heart failure (D:REACH-HF)
Source: Digit Health. 2025 Dec 18;11:20552076251406545. doi: 10.1177/20552076251406545 (PMC12715166; doi:10.1177/20552076251406545)
Supplement: sj-docx-1-dhj-10.1177_20552076251406545 - Supplemental material for Digital adaptation of the clinically effective REACH-HF home-based cardiac rehabilitation programme for people living with heart failure (D:REACH-HF) [file sj-docx-1-dhj-10.1177_20552076251406545.docx]

**Supplementary File** **for** *Digital Adaptation of the Clinically Effective REACH-HF Home-Based Cardiac Rehabilitation Programme for People Living with Heart Failure (D:REACH-HF).*

**van Beurden, Cross, McDonagh, Clark, Thomas, Greaves, Doherty, Taylor & Dalal**

Press Ctrl+Home to get back to this page.

Contents

[**GUIDED Checklist – Guidance for reporting for intervention studies^1^ 2**](#_Toc199515909)

[**TIDieR Checklist – Template for Intervention Description and Replication 3**](#_Toc199515910)

[**SRQR – Standards for Reporting Qualitative Research (SRQR) 5**](#_Toc199515911)

[**Stage 3: Semi-Structured Topic Guide for patient and caregivers** 7](#_Toc199515912)

[**Stage 3: Semi-Structured Topic Guide for healthcare professionals** 11](#_Toc199515913)

# GUIDED Checklist – Guidance for reporting for intervention studies^1^

Duncan E, O’Cathain A, Rousseau N, et al. Guidance for reporting intervention development studies in health research (GUIDED): an evidence-based consensus study. *BMJ Open* 2020; 10: e033516.
<https://www.equator-network.org/reporting-guidelines/guidance-for-reporting-intervention-development-studies-in-health-research-guided/>

|  | **Item description** | **Page in manuscript where item is located** | **Other*** |
| --- | --- | --- | --- |
| **1** | Report the context for which the intervention was developed | Page 4 in Background, Page 5 Design overview | Greaves et al.^2^ |
| **2** | Report the purpose of the intervention development process | Page 4 in Background, Aims & Objectives | Greaves et al.^2^ |
| **3** | Report the target population of the intervention development process. | Page 4 in Background, Aims & Objectives, Page 5 Stakeholder involvement group. |  |
| **4** | Report how any published intervention development approach contributed to the development process | Page 5 Design overview, Page 6 Intervention Planning & Design | Greaves et al.^2^ |
| **5** | Report how evidence from different sources informed the intervention development process. | Page 6 Intervention Planning & Design | Greaves et al.^2^ |
| **6** | Report how/if published theory informed the intervention development process. | Page 6 Intervention Planning & Design | Greaves et al.^2^ |
| **7** | Report any use of components from an existing intervention in the current intervention development process. | Page 6 Original REACH-HF intervention, Intervention Planning & Design | Greaves et al.^2^ |
| **8** | Report any guiding principles, people or factors that were prioritised when making decisions during the intervention development process. | Page 6-8 Intervention planning results,  Table 3 Table of Changes for the D:REACH-HF platform |  |
| **9** | Report how stakeholders contributed to the intervention development process. | Page 5 Figure 1, Stakeholder involvement group, Page 8 Intervention optimisation | Greaves et al.^2^ |
| **10** | Report how the intervention changed in content and format from the start of the intervention development process. | Page 9 - 30 |  |
| **11** | Report any changes to interventions required or likely to be required for subgroups. | Our sample was not able to support a sub-group analysis. |  |
| **12** | Report important uncertainties at the end of the intervention development process | Page 32 |  |
| **13** | Follow TIDieR guidance when describing the developed intervention. | 🗸 |  |
| **14** | Report the intervention development process in an open access format. | 🗸 |  |

*Item has further information regarding the underpinning original intervention here.

# TIDieR Checklist – **Template for Intervention Description and Replication**

Hoffmann TC, Glasziou PP, Boutron I, et al. Better reporting of interventions: template for intervention description and replication (TIDieR) checklist and guide. BMJ 2014; 348: g1687.
<https://www.equator-network.org/reporting-guidelines/tidier/>

|  | **Item description** | **Page in manuscript where item is located** | **Other*** |
| --- | --- | --- | --- |
|  | BRIEF NAME |  |  |
| **1.** | Provide the name or a phrase that describes the intervention. | Page 17 The D:REACH-HF intervention | Greaves et al.^2^ |
|  | WHY |  |  |
| **2.** | Describe any rationale, theory, or goal of the elements essential to the intervention. | Page 17 The D:REACH-HF intervention | Greaves et al.^2^ |
|  | WHAT |  |  |
| **3.** | Materials: Describe any physical or informational materials used in the intervention, including those provided to participants or used in intervention delivery or in training of intervention providers. Provide information on where the materials can be accessed (e.g. online appendix, URL). | Page 17 The D:REACH-HF intervention | Greaves et al.^2^ |
| **4.** | Procedures: Describe each of the procedures, activities, and/or processes used in the intervention, including any enabling or support activities. | Page 17 The D:REACH-HF intervention |  |
|  | WHO PROVIDED |  |  |
| **5.** | For each category of intervention provider (e.g. psychologist, nursing assistant), describe their expertise, background and any specific training given. | Page 17 The D:REACH-HF intervention | Greaves et al.^2^ |
|  | HOW |  |  |
| **6.** | Describe the modes of delivery (e.g. face-to-face or by some other mechanism, such as internet or telephone) of the intervention and whether it was provided individually or in a group. | Page 17 The D:REACH-HF intervention |  |
|  | WHERE |  |  |
| **7.** | Describe the type(s) of location(s) where the intervention occurred, including any necessary infrastructure or relevant features. | Page 17 The D:REACH-HF intervention |  |
|  | WHEN and HOW MUCH |  |  |
| **8.** | Describe the number of times the intervention was delivered and over what period of time including the number of sessions, their schedule, and their duration, intensity or dose. | Page 17 The D:REACH-HF intervention |  |
|  | TAILORING |  |  |
| **9.** | If the intervention was planned to be personalised, titrated or adapted, then describe what, why, when, and how. | Page 17 The D:REACH-HF intervention | Greaves et al.^2^ |
|  | MODIFICATIONS |  |  |
| **10.** | If the intervention was modified during the course of the study, describe the changes (what, why, when, and how). | Page 9 - 30 |  |
|  | HOW WELL |  |  |
| **11.** | Planned: If intervention adherence or fidelity was assessed, describe how and by whom, and if any strategies were used to maintain or improve fidelity, describe them. | NA |  |
| **12.** | Actual: If intervention adherence or fidelity was assessed, describe the extent to which the intervention was delivered as planned. | NA |  |
|  |  |  |  |

*Item has further information regarding the underpinning original intervention here.

# SRQR – Standards for Reporting Qualitative Research (SRQR)

O’Brien BC, Harris IB, Beckman TJ, et al. Standards for reporting qualitative research: a synthesis of recommendations. Acad Med 2014; 89: 1245–1251.
<http://www.equator-network.org/reporting-guidelines/srqr/>

|  | **Item** | **Page/line no(s)** |
| --- | --- | --- |
| Title & Abstract | |  |
| 1 | **Title:** Concise description of the nature and topic of the study Identifying the study as qualitative or indicating the approach (e.g., ethnography, grounded theory) or data collection methods (e.g., interview, focus group) is recommended | This paper reports on more than just the qualitative components and therefore does not refer to this specifically. |
| 2 | **Abstract:** Summary of key elements of the study using the abstract format of the intended publication; typically includes background, purpose, methods, results, and conclusions | 3 |
| **Introduction** | |  |
| 3 | **Problem formulation:** Description and significance of the problem/phenomenon studied; review of relevant theory and empirical work; problem statement | 4 |
| 4 | **Purpose or research question:** Purpose of the study and specific objectives or questions | 4 |
| **Methods** | |  |
|  | **Qualitative approach and research paradigm:** Qualitative approach (e.g., ethnography, grounded theory, case study, phenomenology, narrative research) and guiding theory if appropriate; identifying the research paradigm (e.g., postpositivist, constructivist/ interpretivist) is also recommended; rationale | 5 |
|  | **Researcher characteristics and reflexivity:** Researchers’ characteristics that may influence the research, including personal attributes, qualifications/experience, relationship with participants, assumptions, and/or presuppositions; potential or actual interaction between researchers’ characteristics and the research questions, approach, methods, results, and/or transferability | 21 |
|  | **Context -** Setting/site and salient contextual factors; rationale | 21 |
|  | **Sampling strategy:** How and why research participants, documents, or events were selected; criteria for deciding when no further sampling was necessary (e.g., sampling saturation); rationale | 21 |
|  | **Ethical issues pertaining to human subjects:** Documentation of approval by an appropriate ethics review board and participant consent, or explanation for lack thereof; other confidentiality and data security issues | **21** |
|  | **Data collection methods:** Types of data collected; details of data collection procedures including (as appropriate) start and stop dates of data collection and analysis, iterative process, triangulation of sources/methods, and modification of procedures in response to evolving study findings; rationale | **21** |
|  | **Data collection instruments and technologies:** Description of instruments (e.g., interview guides, questionnaires) and devices (e.g., audio recorders) used for data collection; if/how the instrument(s) changed over the course of the study | **21** |
|  | **Units of study:** Number and relevant characteristics of participants, documents, or events included in the study; level of participation (could be reported in results) | **22** |
|  | **Data processing:** Methods for processing data prior to and during analysis, including transcription, data entry, data management and security, verification of data integrity, data coding, and anonymization/de-identification of excerpts | **21,22** |
|  | **Data analysis:** Process by which inferences, themes, etc., were identified and developed, including the researchers involved in data analysis; usually references a specific paradigm or approach; rationale | **21,22** |
|  | **Techniques to enhance trustworthiness:** Techniques to enhance trustworthiness and credibility of data analysis (e.g., member checking, audit trail, triangulation); rationale | **21** |
| **Results /Findings** | |  |
|  | **Synthesis and interpretation:** Main findings (e.g., interpretations, inferences, and themes); might include development of a theory or model, or integration with prior research or theory | **25-32** |
|  | **Links to empirical data:** Evidence (e.g., quotes, field notes, text excerpts, photographs) to substantiate analytic findings | **25-32** |
|  | **Integration with prior work, implications, transferability, and contribution(s) to the field:** Short summary of main findings; explanation of how findings and conclusions connect to, support, elaborate on, or challenge conclusions of earlier scholarship; discussion of scope of application/generalizability; identification of unique contribution(s) to scholarship in a discipline or field | **32-34** |
|  | **Limitations:** Trustworthiness and limitations of findings | **34** |
| **Other** | |  |
|  | **Conflicts of interest:** Potential sources of influence or perceived influence on study conduct and conclusions; how these were managed | **35** |
|  | **Funding:** Sources of funding and other support; role of funders in data collection, interpretation, and reporting | **35** |
|  |  |  |

**Stage 3: Semi-Structured Topic Guide for patient and caregivers**

| Topic | Questions |
| --- | --- |
| Opening question  First Interview (2-4 weeks)  10-12 weeks | Could you tell me a little bit about how you were trying to manage your heart failure before you started the REACH-HF programme?   - What was your day-to-day life like at the time?  Work, health, family commitments, etc   - Can you talk me through a typical day for you?   - What kinds of activities do you do in a typical day?   - Are there any days or activities that you find more challenging than others?     - Can you tell me a bit more about those?   - In terms of your heart failure, are there good days and bad days?     - Do you do things differently on those days?   - Are there things that you used to do, that you stopped doing because of your heart failure?     - Have you had to make any other adaptations?   Before you started, did you have any expectations of the digital REACH-HF programme?   - If so, what were these? - If not, what were you hoping for at least?   - What did you want to know about?   It’s *** weeks since I last spoke to you, and I wonder if you might be able to tell me what’s been going on for you in that time?   - Since we last spoke, can you tell me if there have been any important changes in your life? *e.g.,* *births, deaths, marriages, other moments of epiphany*   When we spoke before, you were just starting the digital REACH programme, and I’m really interested to know what’s changed for you since then and how this might have impacted you? |
| Engagement with the Intervention First Interview (2-4 weeks)  Second interview (10-12 weeks) | Now you have had access to the programme for a little bit, could you tell me a bit about what REACH-HF is and what it asks you to do?   - For example, if someone you know were to ask you about what it all involves, what would you say to them?   Could you tell me about, if at all, how have you been using the platform?   - **Could you give me an example?**   **What did you think of the programme?**  **How did you get on with using the platform in general?**  Prompts:  How long did it take you to get know how to use it?  How easy /difficult was it to use the platform?  Were things easy to find or not? Could you elaborate and provide me with some examples?  Were there any aspects that were not particularly easy to use or understand?  Were there any parts of the intervention that were especially positive e.g. where you felt things were really working for you?  **Since we last spoke how have you been getting on with D:REACH-HF?**  (Reflect on a topic recalled in that interview if little discussion starts—or something observed)  Has anything changed for you at all? |
| Mediators and Moderators First Interview (2-4 weeks) and Second Interview (10-12 weeks) | **Was there anything outside of the programme (such as work, family or social circumstances) that affected how you got on with D:REACH-HF?**  Prompt:  Were there any barriers you faced to using D REACH-HF? How did this make you feel? Did you overcome these barriers? How did you overcome these barriers?  Is there anything else that affects how you look after your heart?  *Link to answers to first questions about day to day life as prompts here.*   - *Typical daily activities.* - *Any particular challenges caused by HF.* - *Good and bad days.* |
| Involvement of Family and Friends Both interviews | **Do you have any friends or family that help you manage your heart condition?** If so:   Could you tell me a bit more about this?  Did any of them get actively involved in the programme?   - If so, could you give me some examples of this? - If not, could you tell me why?   Was there any shared interaction between you and ** with the facilitator?   - If so, what was that like for you? - If not, why not?   Was *** present in any of the sessions?   - Could you give me an example of what happened in one of these sessions? - What did you think about your (husband, wife, son etc) being present? - If not, why not?   Did you share the Family and Friends resource with them?   - Could you tell me about your decision to get /not get someone involved and share/ or not to share this resource?   - Who did you share it with?   - What is your relationship to this person?   Did you share your Progress Tracker with them?   - Could you tell me a bit how you used it together, if at all? - What did you like or dislike about them getting involved in this? - If not, why not?   What about the chair-based exercises, or the walking programme, did they get involved there at all?   - What were your thoughts about that? - If not, why not?   What, if any, discussions did you have with your family or friends about what support you needed from them in managing your heart failure? |
| Reading the Heart Failure manual contents First interview | **How did you get on with the content of REACH-HF?**  Prompts:  What, if anything at all, did you find helpful?  Were there things that were particularly unhelpful?    **As a result of using the platform and working with your facilitator, what has *changed, if anything at all,* about how you manage your heart failure?**  Prompts:  Do you think REACH-HF has helped you manage your heart condition? If so, could you elaborate on how?  What do you think about the medication section of the manual? What do you think about the physical activity programme? How do you plan to keep your fitness up in the future under your own steam? What other support might be useful? |
| Managing the condition (Progress Tracker) First Interview (2-4 weeks)  Managing the condition (Progress Tracker) First Interview (10-12 weeks) | **What did you think about the progress tracker? Could you describe to me how you used it? If at all, of course?**  **Where there elements that you used more than others?**   - Why?   **What do you do now to look after your heart?**  Prompts:  Do you still exercise?  Do you still self-monitor fluid balance, medication and wellbeing?  Since we last spoke, have you been using your Progress Tracker at all?   - Why and how? - Why not?   **Does this differ from the first interview?**  Prompts: How do you keep track of this?  What other sources of information or support do you use? This is important if there are other heart failure management programmes being used. |
| Relationship with the D REACH-HF facilitator  First Interview (2-4 weeks)  Second interview (10-12 weeks) | Could you describe how the facilitator worked with you?   - What did you like or dislike about how the facilitator worked with you? - Did the facilitator help you identify and meet your needs?   - Could you tell me about how your facilitator helped with this? - Did your facilitator talk about getting someone involved in helping you manage your heart condition?   - What did they say?   Since we last spoke have you had any further contact with the facilitator?   - (explore if yes) - If not, what are your thoughts about that? |
| Processes Both interviews | Has taking part in the D:REACH-HF programme affected your understanding of the condition/your situation?   - If so, could you tell me a bit more about *how* this has impacted your understanding? - If not, could you tell me a bit more about *why* this might be? Is there anything you would have liked/expected from the programme, that wasn’t there?   Has it made you feel any more or less confident about how you manage your heart failure? If so, could you tell me a bit more about *how* this has done this?   - If not, could you tell me a bit more about *why* this might be? Is there anything you would have liked/expected from the programme, that wasn’t there? |
| Psychological adjustment to living with heart failure Both interviews | **Has this programme changed the way you think or feel about having heart failure?**   - If so, in what way? - If not, could you think of any reasons why this might be?   Has this programme changed the way you manage these thoughts or feelings?   - If so, in what way? - If not, could you think of any reasons why this might be?   What do you think about the sections of the manual that are about managing stress?   - Were these helpful? - If so how? Could you tell me more about how you used these? - If not, could you think of any reasons why this might be? |
| D REACH-HF Intervention Adherence First interview (2-4 weeks)  Second Interview (10-12 weeks) | **Do you have any intentions of continuing to use the REACH-HF programme/platform for the remainder of the 12 week programme and beyond?**  Prompt:  Why is this the case? Revisit barriers and facilitators to using the programme.  **Are you still using the REACH-HF platform?**  If no:  When did you stop accessing it?  Could you tell us a bit more about why you stopped accessing it? |
| Further Development of D REACH-HF | If we were to make the REACH-HF – the platform along with facilitation, available to other people with HF, would you change anything? If so, can you describe what you would change and why? |

**Stage 3: Semi-Structured Topic Guide for healthcare professionals**

1. Could you tell me a little bit about your professional background?

Prompt: Types of programmes typically delivered

1. Could you tell me a little bit about the cardiac rehabilitation service you work for?

Prompt: Needs of the patients their service treats. Impact of Covid on the service

1. Could you describe how D REACH-HF was set up in your service and any challenges or facilitators you experienced?

Prompt: Number of staff trained/delivering D REACH-HF, Training received, patient set up, staff set up, adaptations to service to deliver D REACH-HF

1. Before you started what were your expectations of D REACH-HF?
2. Now you have delivered the D REACH-HF programme could you tell me if this has changed?
3. How did you find delivering the D REACH-HF programme?

Prompt: Challenges to delivery, how you overcame challenges, facilitators of delivery, lessons learned

1. How did you go about introducing D REACH-HF to a new patient?

Prompt: How did this differ if at all to the way you introduce REACH-HF? Did this change dependent on the patient, how did you adapt?

1. If present, how did you go about engaging the supporter to the D REACH-HF programme?

Prompts: Barriers, facilitators

1. Could you tell me about the way you used D REACH-HF to support patients?

What did you think of it? How did you use it during the programme with the patients? What did you patients and caregivers think of it?

Specifically ask about

• Progress tracker

• Friend and family resource

• Relaxation audio

• Exercise videos

• Anything else i.e. not part of intervention?

1. Could you tell me how you used the information provided on the facilitator view of D REACH-HF to support delivery of the programme? Did it improve your ability to support your patient?

Progress Tracker

- Fluid Balance
- Exercise
- Medication
- Wellbeing
- Notes.

1. How do you compare delivering REACH-HF (paper based) to D REACH-HF in the digital format?

- Benefits compared to drawbacks
- Differences in patient reception and engagement

1. Moving forward would you continue delivering the DREACH-HF to patients using your service?

If not why not?

1. Moving forward how can we improve the D RECAH-HF platform for use by facilitators and patients?

**Table of Changes for the Digital Adaptation of REACH-HF**

|  | Positive Comments | Negative Comments | Possible Change | Reason For Change * | Agreed Change |
| --- | --- | --- | --- | --- | --- |
| **1. Onboarding** |  |  |  |  |  |
| Onboarding process including accessing, registering, and verification. |  | *[Well looking at that I don't know what it represents [*the REACH-HF logo*]…I don't know what it means.]* |  |  | Not changed – this is the logo for the overarching REACH-HF programme. |
|  |  | Easy to make errors on the phone  [*I can't make it any bigger, its blurred*] | Measures external to the platform put in place (Facilitator to walk through set up with patient) | EAS, REP, EXP(PPI) | Enable adjustable font sizing. |
|  |  | Font and layout of login section is unattractive | Add capacity to change font | EAS, REP, EXP (PPI), NCON | Font made slightly bigger for legibility, but layout remains for now and will be changed in future following feasibility testing if required. |
|  | Participants were fine with having to create passwords  “*That looks fairly standard I think most things, these days are password protected*” Pt 10 v2. | Some participants noted wanting clarity around the criteria /rules for the password.  *[yeah and as long as a forgot password bit…yeah but it usually says forgotten password*.] | Safeguards to be added such as Facilitator retaining access to registration codes.  Provide clarity about the rules for the secure password e.g., how many characters, what type of characters, etc | EAS | Add clear instructions for password creation.  Ensure process for password recovery/reset is presented under the sign-in button. |
|  | Some wanted a way to return to onboarding screens later in case they needed more guidance at a later time. | Some interviewees wanted the ability to skip onboarding entirely | Add a “Skip Introduction” button for users who want to move ahead quickly.  Allow users to return to onboarding screens later via a settings or help menu. | EAS, NCON, | Add a skip introduction button and add a way to navigate back in the side menu. |
| **2. Navigation, functionality, aesthetics and accessibility** | | | | | |
| Navigation | [*I don't mind which option is available, as long as it's clear, and this is very clear.]*  Many participants liked the v2 screen to screen/section to section navigation | Some participants were getting stuck and didn’t know how to move back to where they were. Missing back buttons in v1 | Check screens have back buttons. | EAS, REP, EXP | Update navigation by removing some of the unnecessary steps in navigating between the bigger sections such as from the manual to the progress tracker. |
|  | Participants liked having a quick menu bar to be able to navigate to sections that were anticipated to be frequently used.  *[that's fine, you just have to… you can't have a giant index]* | Some participants were getting confused about where they would look for a specific button as there were multiple menus with repeated navigation buttons. | Check menus for overlap | REP, IMP, EXP | Add clear icons to the “quick menu” at the bottom and remove duplications between menus. |
|  |  | *[Oh yes, I didn't really look at the circles, yes, when you got the circles and the first ones filled in yes that's clear yeah yeah*.] v1 | Use chevrons instead of circles to indicate a section has multiple slides to look at. | EAS, NCON | Replace content completion progress specifier’s current dots with chevrons. |
| Functionality |  |  |  |  |  |
| *Medication* |  | Medication function is limited for some because it is weekly not daily, but others think it’s too often.  *[It needs to be daily. Because…I can't remember when I took all my meds on Sunday]* | Allow preferential choice of medication tracking to be discussed with facilitator | REP, EXP (Participants and PPI), | External daily tracking to be discussed with patient by facilitator, with a weekly check box on the platform. |
| *Progress and Symptom monitoring* | [*Yes, it's good because it makes you think. And nice simple wording. But I suppose it would make you think if you hadn't looked after yourself as well. Like if you saw I haven't had any time to manage my stress obviously that'll be a flag.*]  *[So I'm quite familiar nothing, nothing new really but nice and simple. Daily weekly, monthly that's yes good. Good and straightforward]* | It wasn’t always clear to participants what some of the icons indicated. In v1  It wasn’t always clear to participants what some of the icons indicated.  [*I'm not sure if I was looking at this, I wouldn't really know what am I doing on here, I don't know*.]  *[How are you supposed to know that tick at the side of the blue thing is that to do with fluid intake? Are you gonna, so you've got to sort of go into your app every time you have a drink of water and enter How much you've drunk*.] | Need to find clearer icons. | EAS, IMP, REP, EXP | Co-design suitable icons with the PPI group add way for people to click on the item to see information about what it means – like a hoover over or on click/touch when used on tablet. |
|  |  | Progress monitoring is triggering negative thoughts.  [*I wouldn't want to focus on all this stuff every single week because it would just remind me, you know, of the inevitable end dates which I'm not thinking about too much at the moment.*] | Clear instructions to why progress monitoring is important. Ensure good facilitation around symptom monitoring. | IMP, EXP | Add clear text instructions to why progress monitoring is important. This is also enabled throughout the 12-week programme by the facilitator. |
|  | *[looking at that screen I would expect if I if I click on exercise, for example that I can I can log some exercise*.] | It wasn’t always clear what type of data entry was intended where. For example, there was confusion around where exercise targets were added vs where completed exercise was added.  *[So what happens when you. When you click on that button or oh so the add, add button just before you do that, so the add button does that add just chair-based or complete, no okay*.] | Use labels to indicate setting targets or logging completed exercise. | IMP, REP, EXP | Add labels to indicate setting targets or logging completed exercise on the exercise tracker. |
|  |  | Some found it difficult to see how or where you could edit exercise goals.  *[I guess to edit you can go into notes. You click on each of those*.] | Make navigation for editing exercise target clearer. | EAS, IMP | Add edit button |
|  |  | *[And I'm confused because you've got chair based next. To that, which indicates that that needs to be logged separately, but then, when you go to the plus you didn't have the option chair based or walking*.] | Ensure consistency in data entry labels for exercise target and options of completed exercises. | EAS, IMP | Add the options to the data entry section with clear instructions. |
|  |  | *[You should measure walking speed when you're walking. Right, that seems quite complicated to me, but maybe that's because I've never done it*.] | Link directly to the exercise programme section where this is explained. | IMP | Link directly to the exercise programme section where this is explained.  Ensure health professional training materials covers enabling patients to engage with this through facilitation. |
|  |  | *[Do you have the, can you do metric or so because I personally I've been brought up with kilos so stone and pounds I've no idea is that*.] | Add option to choose between metric and imperial | EAS, EXP | Add option to select metric or imperial. |
|  |  | Medication tracker function is limited for some because it is weekly not daily, but others think daily is too often. v2 | Enable option to add medication adherence on a daily basis instead so this can be left to patient preference. | REP, CONT | External daily tracking is to be discussed with patient by facilitator, as covered in the HF manual, however the platform will only retain a weekly check box to reduce user burden, particularly for those where medication adherence is not a concern (i.e., those who take their medications as prescribed). |
|  |  | The weekly review section about wellbeing (beyond the symptom monitoring section was not clear to all). | Change wording /title and clarify with descriptor text. | EAS, REP, EXP | Adjust wording with stakeholder group. |
|  | Liked seeing the graphs and their progress over time. Some also noted the possibility of using an additional indicator of showing steady progress, to avoid overdoing things.  *[and actually it will be quite nice to have that as a graph so you can watch you going up or you get something...And one of the things they talked about a lot is that doing too much then not being able to do anything. So you get the graph that goes like that...See over activity...because that's not at the moment that hasn't come up anywhere on the APP, and yeah and it's actually pretty good having that, as … having your graph and saying you know you want to achieve is a steady line you don't want to go to this overachievement and inability graph*.] |  | Insert additional line to indicate steady progress to reinforce learning about pacing oneself as covered in the manual. | IMP, EXP, | Graph is not feasible within this project, a steady progress may not be the same for all, and can be very contextual. This requires more expert and clinician input to be able to design.  Add link to sections in HF manual to emphasise scheduling and pacing. This is also externally dealt with via facilitation. |
|  | Feedback in the weekly review about wellbeing was well-received as this cross-referenced to specific sections in the HF manual.  “*Yes, which is really good actually. I like the fact that it's based on what you're putting in so if I'm putting I'm not sleeping on feeling quite low, then it tells me that are there's the manual that can give you some help and advice, so I find that really handy. Rather than you're just putting in icons. And that's it doesn't take you anywhere else*.” |  |  |  |  |
| *Quizzes* | [*Simple to use very self explanatory*] | *Yes, at the end of the thing [answers to the quiz], I see how silly I am* | Add written feedback to indicate correct and incorrect answers rather than just overarching score. | EAS, IMP, REP | Indicate R/W with correct answer then refer to the section (non navigational). Then at the end give a score with questions and a navigational link. |
|  | Buttons at the footer are useful |  |  |  |  |
| *Note sharing between interfaces* | [*For me, if there was a way of communicating where I didn't like something like an email or a text message, or something like that, where I could tell somebody something which wasn't a panic… and they could just read it when they've got some time. Yeah That would be good.*] |  |  |  |  |
| *Favouriting of sections* |  | Being able to favourite specific sections of content to tailor the HF manual to their own needs was considered unnecessary by some as it was easy enough to navigate through the platform  *[Well it's certainly not something I would use if it's only four clicks to get to whatever I'm after*.] | Embed the favouriting of particular content as part of the health professional’s facilitation for the personal-tailoring of the programme. With this tailoring, use this tailored section as the default view. However, it needs to be made clear to the patient through facilitation and on the platform itself, that they can still access the rest of the manual at any point. For example, when priorities change or the patient just wishes to explore further. | EXP | Present list of favourites that can be changed in order at any time. |
|  |  | Others did like it but had barriers in terms of how the favouriting worked and was displayed.  *[yeah well, I mean why, why are things by date...I would just expect to see a list of my favourites...I’m not going to go into I think oh three weeks ago I put something in that I wanted to see.. and I think the other thing is with it is to be able to move things around so as you are, you can prioritize them, so the ones use a lot, you can put at the top of the list and the ones you occasionally use, you can put at the bottom of the list*.] v2 | Enable re-ordering of favourited sections as opposed to only ordering by the data it was favourited. Present as a list of overarching sections, rather than specific pages favourited as this takes the page out of context. | IMP, EXP | Not feasible in the scope of this current project, further co-design work with stakeholders required. |
| Aesthetics and Accessibility | Diagrams at the beginning of the manual are good quality “*that's nice I like the colour like the heart.* [F02]” |  |  |  |  |
|  |  | Participants didn’t like the purple background in v1 | Use white for a cleaner aesthetic | EAS, REP, EXP | Change purple background for white and adjust text and image colours to retain suitable contrast for accessibility. |
|  |  | Images and icons in the rest of the manual are poor quality | Improve quality/resolution of images | EAS, REP, EXP (PPI), NCON | Improve quality of images |
|  |  | Text is too small “Well I can barely read it” | Enable capacity to increase text size | EAS, REP, EXP (PPI and participants), NCON | Add options to change font sizes in account settings |
|  |  | [*Right and it's hard to see the grey on white I can read the read in the blank but the Gray, is just not and there's not enough depth connection between the two.*] | Increase contrast and enable high contrast option for increased accessibility. | EAS, REP, EXP (PPI and participants), NCON | Check contrast and legibility and increase contrast. Also add in a high contrast option (black and yellow) accessible through account settings. |
|  | Layout makes it easy to read  “*yeah that's simple...that's clear it's not too fussy not too wordy it's just yeah*” |  |  |  |  |
|  |  | Information in places is seen as unengaging and longwinded. | Better to have more bite sized chunks of information - more bullet points, photos, diagrams, animations, videos to make it more memorable and interactive. Have a recap at the end of each section and a summary at the beginning | EAS, REP, IMP, NCON | Text broken up into smaller paragraphs and with imagery where possible. |
|  |  | Information is not much fun | More photos, diagrams, animations, videos to make it more memorable and interactive | EAS, REP, IMP | Text broken up by images and graphics. |
|  | The text boxes are an engaging way to present information |  |  |  |  |
|  |  | Needs to be suitable for different learning styles | Ensure incorporation of mixed delivery of content. Written info, quizzes, videos | REP | Text broken up with images, quizzes and videos. |
| **3. Content** | | | | | |
| Written elements from the REACH-HF manuals. |  | Wording is didactic [*How about you and your facilitator may discuss the main priorities. Rather than will. That sounds a bit. School master ish...soften that 'will' word... that first line is a bit bit harsh I'd say*.] | Use could rather than should | EAS, IMP, REP, EXP (PPI and literature), | None agreed – wording remains the same as this is copyright IP in manual-based REACH-HF and will require agreement from IP holders. |
|  | [*definitely good you don't hear much about power really when you have heart problems you just hear the negative. I like the way uses it positively that's good*] |  |  |  |  |
|  |  | [*Yes, at the end of the thing is, I see how silly am]* | Text based summary at the end of quizzes to indicate correct or incorrect answer. | EAS | Indicate correct or incorrect during the quiz, and refer to the related educational section on the platform.  At the end of the quiz give an overall score with a list of the questions and a navigational links to the related educational sections on the platform. |
|  |  |  |  |  |  |
| **4.Facilitation** |  |  |  |  |  |
|  | [*Yeah okay well that that's that would be very useful. If, its healthcare professional if that's the right description. Rather than a database management.*] |  |  |  |  |

N.B: *IMP - Important for Behaviour Change; EAS - Easy and uncontroversial; REP – Repeatedly; EXP – Experience; NCON – Does not contradict; CONT - Contradict
